# Multiple DSB Resection Activities Redundantly Promote Alternative End Joining-Mediated Class Switch Recombination

**DOI:** 10.3389/fcell.2021.767624

**Published:** 2021-11-26

**Authors:** Xikui Sun, Jingning Bai, Jiejie Xu, Xiaoli Xi, Mingyu Gu, Chengming Zhu, Hongman Xue, Chun Chen, Junchao Dong

**Affiliations:** ^1^ Department of Immunology, Zhongshan School of Medicine, Sun Yat-sen University, Guangzhou, China; ^2^ Key Laboratory of Tropical Disease Control (Sun Yat-sen University), Ministry of Education, Guangzhou, China; ^3^ Department of Gastroenterology, the Third Affiliated Hospital of Sun Yat-sen University, Guangzhou, China; ^4^ Research Center of the Seventh Affiliated Hospital, Sun Yat-sen University, Shenzhen, China; ^5^ Department of Pediatrics, the Seventh Affiliated Hospital of Sun Yat-Sen University, Shenzhen, China

**Keywords:** DNA double-strand breaks repair, alternative end joining, class switch recombination, DSB end resection, microhomology

## Abstract

Alternative end joining (A-EJ) catalyzes substantial level of antibody class switch recombination (CSR) in B cells deficient for classical non-homologous end joining, featuring increased switch (S) region DSB resection and junctional microhomology (MH). While resection has been suggested to initiate A-EJ in model DSB repair systems using engineered endonucleases, the contribution of resection factors to A-EJ-mediated CSR remains unclear. In this study, we systematically dissected the requirement for individual DSB resection factors in A-EJ-mediated class switching with a cell-based assay system and high-throughput sequencing. We show that while CtIP and Mre11 both are mildly required for CSR in WT cells, they play more critical roles in mediating A-EJ CSR, which depend on the exonuclease activity of Mre11. While DNA2 and the helicase/HRDC domain of BLM are required for A-EJ by mediating long S region DSB resection, in contrast, Exo1’s resection-related function does not play any obvious roles for class switching in either c-NHEJ or A-EJ cells, or mediated in an AID-independent manner by joining of Cas9 breaks. Furthermore, ATM and its kinase activity functions at least in part independent of CtIP/Mre11 to mediate A-EJ switching in Lig4-deficient cells. In stark contrast to Lig4 deficiency, 53BP1-deficient cells do not depend on ATM/Mre11/CtIP for residual joining. We discuss the roles for each resection factor in A-EJ-mediated CSR and suggest that the extent of requirements for resection is context dependent.

## Introduction

Mature B cells undergo immunoglobulin heavy chain (*IgH*) class switch recombination (CSR) to mediate different antibody effector functions. CSR replaces the initially expressed µ constant gene (Cμ) with a downstream constant gene through genomic DNA recombination (Xu et al., 2012). In the mouse *IgH* locus, six independently transcribed C_H_ genes, Cγ3, Cγ1, Cγ2b, Cγ2a, Cε, and Cα, line up to 200 kb downstream of Cμ. A long and repetitive intronic switch region (4–12 kb) with tandem G-rich repeat sequences on the non-template strand lies between each C_H_ gene and its I promoter. Stimulating B cells with combinations of activators and cytokines directs CSR to particular C_H_ genes by modulating germline transcription to recruit AID, which introduces into S regions multiple C to U mutations that are subsequently converted to staggered double-strand breaks (DSBs) by base excision and mismatch repair with yet unclear mechanisms (Hwang et al., 2015; Yu and Lieber, 2019). CSR is completed by joining donor Sμ and acceptor S region DSBs in a deletion-preferred fashion to promote antibody production (Dong et al., 2015).

AID-initiated S region DSBs are efficiently repaired by the classical non-homologous end joining (c-NHEJ) pathway, which simply aligns and religates two broken ends with minor modification. Ku/DNA-PKcs and Lig4/XRCC4 complexes are the core components of c-NHEJ and depletion of any of these factors in mature B cells significantly, but not completely reduces CSR efficiency (Boboila et al., 2010; Boboila et al., 2012). In fact, CSR to IgG in cells deficient for Ku, Lig4, or both can still occur at levels to ∼30% of WT cells with altered kinetics, strongly implicating alternative end joining (A-EJ) pathways for residual switching (Yan et al., 2007; Boboila et al., 2010). Sanger and high-throughput sequencing of the junctions of residual Sμ-Sx joins revealed elevated usage of microhomology (MH) sequences (usually 1–5 bp in length) shared between donor and acceptor DSBs in the absence of Ku and/or Lig4, indicating that A-EJ preferred microhomology-mediated end joining (MMEJ). It is noteworthy that MH represents a significant feature but does not serve as a defining factor for A-EJ, as NHEJ repair in WT cells also utilizes MH in a significant portion of junctions. It has been proposed that PARP1 and the Lig3/XRCC1 complex are requisite A-EJ factors (Frit et al., 2014). Early evidence supporting this notion came from ligation of DNA substrates with protruding overhang ends (Vogel et al., 2003; Audebert et al., 2004). However, *in vivo* study with activated primary B cells only revealed a rather minor role for PARP1 in MH usage and no impact on IgG switching efficiency *per se* (Robert et al., 2009). In addition, conditional knockout of XRCC1 in both WT and Lig4-deficient B cells did not affect either CSR or chromosomal translocations (Boboila et al., 2012). The latter finding raised the possibility that DNA ligase I also plays a role in A-EJ, which was supported by later studies that deleting either nuclear Lig3 or Lig1 in Lig4-deficient CH12F3 cells conferred no additional CSR defect than Lig4 deletion alone. As mammals only have these three ligases, this suggests that Lig1 and Lig3 are redundant in A-EJ (Lu et al., 2016; Masani et al., 2016). As Lig1 and nuclear-form Lig3 deletion alone in WT did not render the cells obvious defect in end joining and CSR (Han et al., 2014; Masani et al., 2016), whether and how A-EJ occurs in WT cells are currently difficult to assess and awaits more careful dissection. AID-initiated S region DSBs also trigger activation of DNA damage response (DDR) kinase Ataxia telangiectasia-mutated (ATM), which phosphorylates a series of downstream substrates including histone variant, H2AX, MDC1, 53BP1, etc., that assemble into macromolecular foci surrounding DSBs to amplify damage signals and tether DSB ends for efficient repair (Xu et al., 2012). Deficiency for DDR factors has been shown to severely impair end joining during V(D)J recombination (Helmink et al., 2011; Zha et al., 2011; Liu et al., 2012; Oksenych et al., 2013) and leads to impaired CSR at 30–50% of corresponding wild type cells and accumulation of substantial AID-dependent *IgH* breaks, indicating a role for ATM/H2AX in the joining phase of CSR (Reina-San-Martin et al., 2004; Franco et al., 2006; Boboila et al., 2012). Ablation of 53BP1 results in the most profound CSR defect where only about 5% of wild type switching level is observed accompanied by increased intra-S joining and *IgH* specific break burden (Manis et al., 2004; Ward et al., 2004; Reina-San-Martin et al., 2007; Bothmer et al., 2011). Recently, Rif1 has been identified as a phosphor-53BP1-associating effector protein that suppresses DSB resection, a 5’->3′ nucleolytic process to expose 3′ single-stranded overhangs at broken ends (Escribano-Díaz et al., 2013; Zimmermann et al., 2013); accordingly, Rif1-deficient cells display largely impaired CSR to downstream S regions (Callen et al., 2013; Chapman et al., 2013; Di Virgilio et al., 2013). In this regard, ATM-dependent DDR has been shown to promote c-NHEJ during CSR at least in part by preventing extensive S-region DSBs end resection and MMEJ (Yamane et al., 2013; Dong et al., 2015; Panchakshari et al., 2018).

It has been well documented that 5’->3′ DSB end resection is required for homologous recombination (HR) and MMEJ of DSB repair in yeast and higher eukaryotes (Symington, 2016). While HR requires longer homology to the sequence around DSB ends for base pairing, MMEJ may, in principle, involve shorter resection to expose MH sequences for annealing. DSB resection is initiated by the coordinated action of DNA nuclease complex MRN and CtIP. MRN complex consists of RAD50, NBS1, and Mre11 that renders the complex endonuclease and 3′-5′ exonuclease activity, an orientation opposite to the ongoing resection (Garcia et al., 2011). Recent study revealed that Mre11 uses its endonuclease activity to nick DNA at 3′ downstream vicinity of DSB and its exo-activity to degrade DNA strand towards the break to expose single-stranded DNA (Paull, 2018). While Mre11 has been shown to be critical for both c-NHEJ and A-EJ-mediated CSR (Dinkelmann et al., 2009), the exact role for CtIP in CSR is less clear (Lee-Theilen et al., 2011; Bothmer et al., 2013; Liu et al., 2019). Human *CtIP* encodes a 5′-flap endonuclease on branched DNA structure that participates in resection initiation mainly by stimulating Mre11’s endonuclease activity independent of its endonuclease activity (Sartori et al., 2007; Makharashvili et al., 2014), and CtIP phosphorylation at T855 by ATM is critical for its role in resection (Peterson et al., 2013; Wang et al., 2013). A recently identified exonuclease EXD2 has been shown to functionally interact with MRN to accelerate DSB resection with its 3′-5′ exonuclease activity and is required for efficient HR (Broderick et al., 2016), but its role in MMEJ/A-EJ remains to be exploited. After Mre11/CtIP-mediated initiation to degrade up to hundred nucleotides close to the break, helicase BLM/WRN and endonuclease DNA2 switches on to promote long range resection up to tens of kilobases away from the break, and this activity appears redundant with exonuclease Exo1 (Symington, 2016).

The observation that both *lig4* ^*−/−*^ and *53bp1* ^*−/−*^ cells exhibit greatly increased DSB resection and similarly elevated MH usage in Sμ-Sx junctions raised the question of which activities are involved in S region DSB resection in these cells and whether DSB resection accounts for all or part of CSR defect. In this regard, thorough investigation on the role for DSB resection in A-EJ-mediated CSR is still lacking. In this study, we systematically examined the requirements for each individual protein involved in DSB resection machinery in activated B cells proficient or deficient for Lig4 or 53BP1. Our results revealed that resection factors play important roles in A-EJ mediated CSR, and ATM kinase activity with CtIP/Mre11 in A-EJ. In addition, although both Lig4 and 53BP1 deficiency lead to c-NHEJ defect with remarkably similar MH patterns (Panchakshari et al., 2018), their need for DSB resection to assist residual joining varied greatly. In summary, our work indicated that B cells harness multiple DSB resection activities to engage A-EJ-mediated CSR in a context-dependent manner.

## Materials and Methods

### Cell Culture

All of CH12F3 cell lines in this study were cultured with in RPMI 1640 (10-040-CV, Corning) supplemented with 15% FBS (FSP500, ExCell Bio), 100 mΜ β-mercaptoethanol (0482-250 ml, Amresco), 20 μM HEPS (25-060-CI, Corning), 2 mM L-Glutamine (25-005-CI, Corning), 1× MEM non-essential amino acid (25-025-CI, Corning), 1 mM sodium pyruvate (25-000-CI, Corning), 1× penicillin streptomycin (30-002-CI, Corning). 293T and Phoenix Ampho were maintained in DMEM (10-013-CV, Corning) supplemented with 10% FBS (ExCell Bio) and 1× penicillin streptomycin.

### Plasmids

PSpCas9(BB)-2A-GFP (pX458) plasmid was obtained from Addgene (#48138). All the gRNA oligonucleotides were cloned into pX458. All the oligonucleotides sequences were listed in Appendix information, Supplementary Table S1. pMSCV-IRES-GFP II (pMIG II) plasmid was obtained from Addgene (#52107). pLKO.1 puro plasmid was obtained from Addgene (#8453). pMD2.G and psPAX2 plasmids were kindly gifted by the F.W.A. laboratory.

### Construction of Gene Knockout Cell Lines.

The gene deletion strategies were performed according to the essential domain of genes reported (Taccioli et al., 1998; Babbe et al., 2009; Schaetzlein et al., 2013; Broderick et al., 2016; Panchakshari et al., 2018; van Wietmarschen et al., 2018). WT CH12F3 cell line or its mutants were nucleofected with a pair of pX458 vector with two gRNAs flanking one or two exons using the 4D Nucleofector Kit (solution SF, protocol CA-137; Lonza). At 24–48 h post-nucleofection, the GFP positive cells were sorted with Beckman Coulter MoFlo Astrios EQs and plated into 96-well plates. Single cell clones were marked and screened by PCR. Positive clones were further confirmed by western blot analysis or T-A cloning and sequencing.

### Antibody

The primary antibodies used in this study were as follows: anti-ATM Rabbit antibody (D2E2, #2873, Cell Signaling Technology), anti-γ-Tubulin antibody (#5886, Cell Signaling Technology), anti-Mre11 Antibody (#4895, Cell Signaling Technology), anti-DNA-PKcs (G-12, SC-390849, Santa Cruz), anti-CtIP (D-4, SC-271339, Santa Cruz), anti-EXD2 antibody (20138-1-AP, Proteintech), anti-phospho KAP1 (S824) antibody (A304-146A-M, Bethyl Laboratories), anti-β-Actin antibody (66009-1-Ig, Proteintech), anti-AID monoclonal antibody (mAID-2, 14-5959-82, eBioscience), and anti-Flag M2 antibody (F1804-50UG, Sigma-Aldrich). The antibodies for flow cytometry analysis were anti-Mouse lgM-APC (17-5790-82, eBioscience), anti-Mouse lgA-PE (12-4204-83, eBioscience), and anti-Mouse lgG1-PE (406608, Biolegend).

### Chemicals and DNA Damaging Treatments

Mirin (M9948-5 MG, Sigma-Aldrich), PFM01 (SML1735-5mg, Sigma-Aldrich), Ku55933 (SML 1109-5 mg, Sigma-Aldrich), and AZD1390(S8680-5 mg, Selleck) were dissolved in DMSO and stored at –20°C. Cells were exposed to X-rays generated by a Rad Source RS2000 Irradiator (160 kv, 25 mA) to induce DNA damage.

### Short Hairpin RNA-Mediated Gene Silencing

ShRNAs specific to Mre11, CTIP, and DNA2 were cloned into pLKO.1 puro vector. All the shRNA sequences were listed in Appendix information, Supplementary Table S1. The plko.1 vector cloned with specific shRNA sequence and the packaging plasmids pMD2. G and psPAX2 were co-transfected into HEK293T cell to produce lentiviruses with polyethylenimine (PEI) transfection reagent. Cell supernatants were collected after 48 h post-transfection and filtered with a sterile 0.45-µm syringe filter to remove cell debris. The lentiviruses were concentrated by virus precipitation Solution (ExCell Bio) and resuspended in complete medium. CH12F3 or its mutants were infected with lentivirus by centrifuging at 32°C 1,000×*g* 60 min. After transduction for 48 h, the cells were selected with 0.5 μg/ml puromycin for 5–7 days.

### EXO1^WT/^EXO1^EK^ Rescue Experiment

The EXO1 cDNA sequence was obtained using reverse transcription from total RNA extracted from WT CH12F3 cell line. EXO1 mutants EXO1^EK^ was obtained with site-directed mutagenesis. The C terminus of cDNA was added with 3× flag tag by two sequential PCR rounds. The EXO1/EXO1^EK^-3× flag were cloned into pMIG II. Retrovirus vector pMIG II-EXO1^WT^/EXO1^EK^-3× flag were transfected into Phoenix Ampho cell to produce retrovirus with PEI. Retrovirus was concentrated as lentivirus did. EXO1-deficient CH12F3 were transduced with retrovirus by centrifuging at 32°C 1,000×*g* for 60 min. After transduction for 3–4 days, the GFP positive cells were sorted with Beckman Coulter MoFlo Astrios EQs. To confirm the expression of EXO1^WT^/EXO1^EK^ in EXO1-deficient CH12F3 was rescued, the infected cells were lysed for western blot analysis with anti-flag primary antibody.

### Class Switch Recombination Assay

WT CH12F3 cell line or its mutants at a density of 5 × 10^4^ cells/mL or 1 × 10^5^ cells/mL were stimulated with 1 μg/ml anti-CD40 (16-0401-86, eBioscience), 20 ng/ml IL4 (214-14, PeproTech), and 1 ng/ml TGF-β (96-100-21-10, PeproTech) for 72 h. Cells were collected and analyzed by flow cytometry. Data were presented as mean ± SD from independent experiments (Student’s *t*-test, *p < 0.05, **p < 0.01, ***p < 0.001, ****p < 0.0001, n. s indicates non-significant differences).

### Cas9-Initiated Class Switch Recombination Assay

For CRISPR/Cas9-initiated CSR (Cas-CSR) in CH12F3 cells, sgRNAs targeting up- and down-stream S regions (Sμ and Sγ1) were transfected into CH12F3 cells via electroporation. SgRNAs were cloned into px458 plasmids. After transfection, CSR to IgG will increase gradually. CSR level to other Ig in KO cells was normalized to the GFP + ratio of 24 h after transfection.

### Western Blotting

Cells were collected and lysed in RIPA buffer with fresh proteinase inhibitors. The cell lysate was centrifuged and quantified by the BCA assay (23225, Thermo). The collected cell lysate was denatured by boiling in loading buffer at 100°C for 10 min, loaded into the wells of SDS/PAGE to separate, and transferred to PVDF membranes (IPVH00010, Merck). The membranes were blocked by 5% skim milk in PBST for 1 h at room temperature, probed with indicated primary antibodies overnight at 4°C, washed 3 times with PBST, incubated with recommended HRP-conjugated second antibody (7074s, Cell Signaling Technology) for 1 h at room temperature, washed 3 times with PBST, and visualized with HRP substrate peroxide solution.

### Quantitative RT-PCR

Total RNA was extracted using TRIzol reagent (15596026, Invitrogen). RNA was reverse transcribed into cDNA by the reverse transcription system (RR037A, Takara). SYBR Premix Ex Taq kit (RR820A, Takara) was used to perform qRT-PCR on LightCycler480 Real-Time PCR System (Roche). Relative gene expression levels were obtained based on the 2^−∆∆Ct^ method with Hprt as internal reference control. Primers for qRT-PCR are listed in Supplementary Table S1.

### HTGTS

HTGTS libraries were constructed as described (Dong et al., 2015). Briefly, genomic DNA of CH12F3 or its mutants were extracted after stimulation for 3 days. The genomic DNA was sonicated and amplified by LAM-PCR with 5′ Sμ biotin primer (5′-CAG​ACC​TGG​GAA​TGT​ATG​GT-3′). The Biotinylated products of PCR were captured by Dynabeads MyOne streptavidin C1 beads (Invitrogen), ligated with bridge adapters on-bead. The ligated products were amplified by second-PCR to add adaptor. Then, the products of PCR were blocking with endonuclease Afill to remove germline genomic DNA fragment. The third round PCR was performed to add Illumina Miseq-compatible adapters to conduct MiSeq sequencing. The HTGTS data were analyzed as described (Dong et al., 2015; Panchakshari et al., 2018). Data were presented as mean ± SEM (Student’s *t*-test, *p < 0.05, **p < 0.01, ***p < 0.001).

### Statistical Analysis

Statistical analysis was performed in GraphPad Prism 7.01. Data was reported as mean and SD except that the HTGTS analysis was reported as mean and SEM. Unpaired two-tailed Student *t* test or two-way ANOVA was used to examine the significant difference between samples. The asterisks stand for significant differences (*p < 0.05, **p < 0.01, ***p < 0.001, ****p < 0.0001, n. s indicates non-significant differences).

### Data and Code Availability

HTGTS sequencing data have been deposited at the Sequence Read Archive (SRA) with a project #PRJNA728565, with an access URL:https://dataview.ncbi.nlm.nih.gov/object/PRJNA728565.

## Results

### Mre11 and CtIP are Required for A-EJ Mediated Class Switch Recombination to IgA

**FIGURE 1:**
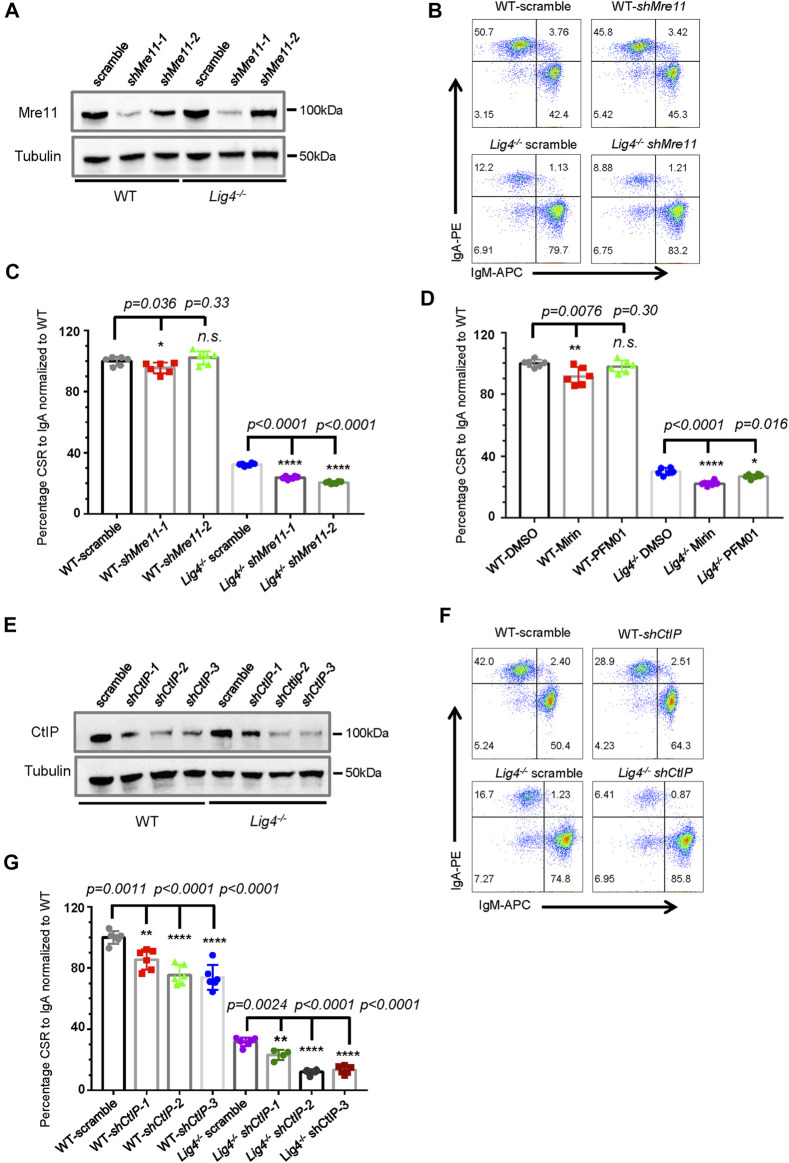
Mre11 and CtIP are essential for A-EJ mediated CSR. **(A)** Western blot analysis of Mre11 expression in WT and *Lig4* ^*−/−*^ CH12F3 cells transduced with lentivirus expressing the indicated shRNA. **(B)** Representative flow cytometry analysis of CSR to lgA in Mre11-silenced WT and *Lig4* ^*−/−*^ cells. **(C)** Quantification of IgA switching efficiency in Mre11-silenced WT and *Lig4* ^*−/−*^ cells normalized to that scramble control. Data were presented as mean ± SD from six independent experiments (Student’s *t*-test, *p < 0.05, **p < 0.01, ***p < 0.001, ****p < 0.0001, n. s. (*p* > 0.05) indicates non-significant differences). **(D)** Normalized CSR to lgA in WT and *Lig4* ^*−/−*^ cells pretreated with 10 μΜ exonuclease inhibitor (Mirin), 10 μΜ endonuclease inhibitor (PFM01). Data were presented as mean ± SD from six independent experiments (Student’s *t*-test, *p < 0.05, **p < 0.01, ***p < 0.001, ****p < 0.0001, n. s. (*p* > 0.05) indicates non-significant differences). **(E)** Western blot analysis of CtIP expression in WT and *Lig4* ^*−/−*^ cells transduced with lentivirus expressing the indicated shRNA. **(F)** Representative flow cytometry analysis of lgA switching in CtIP-knockdown WT and *Lig4* ^*−/−*^ cells. **(G)** Quantification of lgA CSR efficiency in CtIP knockdown WT and *Lig4* ^*−/−*^ cells. CSR was assayed at 72 h after stimulation with α-CD40/IL-4/TGF-β. Scramble represented control shRNA targeting a non-mouse sequence. Data were presented as mean ± SD from six independent experiments (Student’s *t*-test, *p < 0.05, **p < 0.01, ***p < 0.001, ****p < 0.0001, n. s. (*p* > 0.05) indicates non-significant differences).

Previous reports indicated that germline deletion of Mre11 or CtIP confers early embryonic lethality in mice, and mutant MEF cells showed altered proliferation and genome instability (Buis et al., 2008; Chen et al., 2005). To first examine the role of Mre11 in class switching, we utilized two different short hairpin RNAs (shRNA) expressed from lentiviral vectors to silence its expression in mouse mature B cell lymphoma cell line CH12F3 that can be stimulated to specifically undergo isotype switching to IgA (Figure 1A). ShRNA-mediated knock down of Mre11 expression in CH12F3 appears not affecting the overall proliferation of cells (Supplementary Figure S1A). When stimulated by the combination of αCD40/IL-4/TGF-β, shMre11 cells showed similar level of mature Iμ and Iα germline transcription, and the protein level of AID was not perturbed by Mre11 silencing (Supplementary Figures S1B,S1C). IgA expression in shMre11 cells showed a mild defect by surface staining (Figures 1B,C, Supplementary Figure S2A). To distinguish whether the endonuclease or exonuclease of Mre11 is involved in CSR by c-NHEJ, we treated CH12F3 cells with small chemical inhibitor Mirin or PFM01 that specifically inhibit Mre11’s exo- or endonuclease activity, respectively (Shibata et al., 2014), and discovered that only Mirin, but not PFM01 treatment conferred a mild but significant defect in IgA levels (Figure 1D, Supplementary Figure S2B). Next, we used three different shRNA to silence expression of CtIP in CH12F3 cell (Figure 1E). While CtIP knockdown did not affect the Iμ and Iα germline transcription, AID protein level and cell proliferation rate did exhibit small decline by shCtIP-3# (Supplementary Figures S1D-F). However, all three shCtIP-infected cells showed similar IgA levels at around 70–80% of values of WT, implying that CtIP contributes to class switching in WT cells largely independent of AID protein regulation. A recent study reported EXD2 as an exonuclease that functions with Mre11 for DSB resection and HR (Broderick et al., 2016). We generated EXD2 knockout CH12F3 cells by CRISPR/Cas9 (Supplementary Figures S3A–S3C), and surface staining indicated that EXD2 was not required for IgA switching by c-NHEJ (Supplementary Figure S3D).

To further investigate whether these resection initiation proteins are required for CSR by A-EJ, we knocked-down Mre11 in *Lig4* ^*−/−*^ CH12F3 cells (Figure 1A). Again, Mre11 silencing in *Lig4* ^*−/−*^ cells did not change Iμ/Iα germline transcription and AID protein level, or overall proliferation (Supplementary Figures S1A–S1C). As previously reported, *Lig4* ^*−/−*^ cells switched to IgA at an efficiency about ∼30% of that of WT CH12F3 cells. Mre11 silencing by two different hairpin RNAs significantly further reduced IgA levels by about one third to half (Figures 1B,C). Treating *Lig4* ^*−/−*^ cells with either Mirin or PFM01 also reduced IgA switching levels to close to 50% of DMSO-treated control cells (Figure 1D, Supplementary Figure S2B). Similarly, shRNA-mediated knockdown of CtIP in *Lig4* ^*−/−*^ cells further impairs IgA switching by more than 50% (Figures 1F,G, Supplementary Figure S2C). A rather mild effect on A-EJ was observed by *Exd2* deletion in *Lig4* ^*−/−*^ cells (Supplementary Figures S3C,S3D), suggesting that the stimulation of Mre11’s exonuclease activity by Exd2 is negligible during B cell class switching. Taken together, these data suggest that while efficient CSR in wild type cells requires Mre11/CtIP to various extent, Mre11 and CtIP play more important roles in A-EJ-mediated CSR.

### S-S Joining Pattern and MH Usage in c-NHEJ and A-EJ in the Absence of Mre11 or CtIP

**FIGURE 2:**
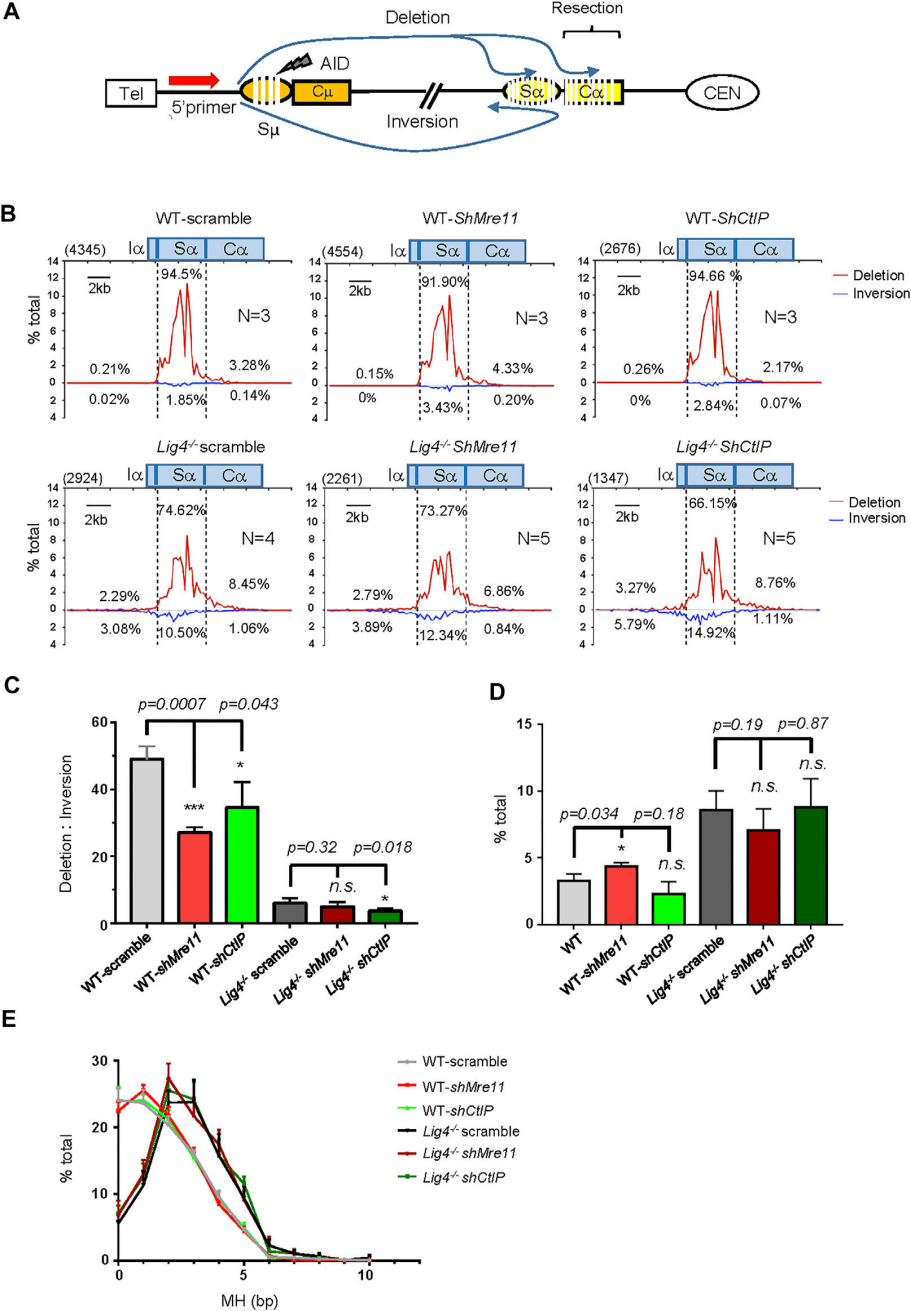
S-S junction profile and MH usage pattern in Mre11/CtIP-silenced WT and *Lig4−/−* cells. **(A)** Diagram of the joining outcomes between Sμ and Sα DSB sequenced by HTGTS. Joining from 5′ Sμ bait DSB to Sα broken end reading in the telomere to centromere orientation are designated as deletion and represent productive joining, whereas those from 5′ Sμ to Sα broken end reading from centromere telomere orientation are designated as inversion and represent non-productive joining. Junctions falling into distal Cα region are denoted as long resection. **(B)** Linear distribution of pooled Sμ-Sα junctions recovered from Mre11/CtIP-silenced WT and *Lig4* ^*−/−*^ cells with indicated numbers (*N*) of experiment repeats are shown in the form of deletion or inversion along a 20-kb region centered at core Sα (Chr12: 114491001–114511000). Bin size is 200 bp and 100 bins are presented in each plot. Numbers in the parenthesis represent total unique junctions in the indicated region. **(C)** The ratio of deletion versus inversion for Sα junctions in Mre11/CtIP-silenced WT and *Lig4* ^*−/−*^ cells. Data were presented as mean ± SEM (Student’s *t*-test, *p < 0.05, **p < 0.01, ***p < 0.001, n. s. (*p* > 0.05) indicates non-significant differences). **(D)** Percentage of long resection junctions in Mre11/CtIP-silenced WT and *Lig4* ^*−/−*^ cells. Data were presented as mean ± SEM (Student’s *t*-test, *p < 0.05, n. s. (*p* > 0.05) indicates non-significant differences). **(E)** The MH pattern of Sμ-Sα junctions in Mre11/CtIP-silenced WT and *Lig4* ^*−/−*^ cells. HTGTS analyses were performed with indicated cells stimulated with α-CD40/IL-4/TGF-β for 72 h. Data were presented as mean ± SEM.

To further explore the molecular signature of end joining in cells deficient for Mre11 or CtIP, we utilized High Throughput Genome-wide Translocation Sequencing (HTGTS) to characterize Sμ-Sα junctions and MH usage pattern in stimulated CH12F3 wild type and mutant cells (Figure 2A). HTGTS with a 5’ Sμ anchor primer fine-maps joining from AID-initiated DSBs occurring in upstream Sμ to those in Sα region and genome wide. Consistent with no or moderate defect in c-NHEJ CSR by FACS in WT CH12F3 cells, Mre11 or CtIP knockdown cells showed nearly no difference in the percentage of Sμ-Sα joining compared with scramble controls (Supplementary Figure S4). When examining junctions mapped to the Sα region for details, however, we indeed observed a small but significant decrease in the ratio of direct versus inversional Sμ-Sα joining in shMre11 cells (Figures 2B,C). The percentages of junctions falling into Cα represents joining of Sμ to Sα DSBs resected into distal region (Figure 2A). In shMre11 cells, we observed a small but significant increase in the Sα DSBs long resection (Figure 2D), and MH usage of Sμ-Sα joining showed a slight decrease in “blunt” (MH = 0) and increase in MH = 1 joins (Figure 2E), consistent with a role for Mre11 in activating DDR kinase ATM that is critical for suppressing resection and MH usage. In contrast, CtIP-silenced CH12F3 cells showed identical Sα DSB resection and MH pattern in Sμ-Sα junctions compared with control cells (Figures 2D,E), indicating that CtIP does not play a critical role in the joining step of c-NHEJ-mediated CSR.

We then analyzed S-S joining pattern of Mre11 or CtIP-silenced *Lig4* ^*−/−*^ cells with HTGTS. Scramble control virus transduced *Lig4* ^*−/−*^ cells had significantly decreased in direct Sα joining and concomitant decrease in the ratio of direct versus inversional Sα junctions (Figures 2B–D). Mre11 or CtIP knockdown further decreased direct Sα joining percentage, consistent with IgA surface staining data (Figure 2B). Interestingly, we found no significant difference in Cα distal junctions and MH usage between *Lig4* ^*−/−*^ cells infected with scramble or shMre11/shCtIP virus (Figures 2D,E), indicating that while Mre11/CtIP is partly required for A-EJ events in Lig4-deficient cells, they are not required for the long resection activity into Cα region in these cells. In addition, we indeed discovered an obviously decreased ratio of direct versus inversional Sα junctions in shCtIP infected *Lig4* ^*−/−*^ cells (Figures 2B,C), implicating a unique role for CtIP in A-EJ in this context.

### BLM/DNA2-Mediated Long-Range Resection is Required for A-EJ Class Switch Recombination

**FIGURE 3:**
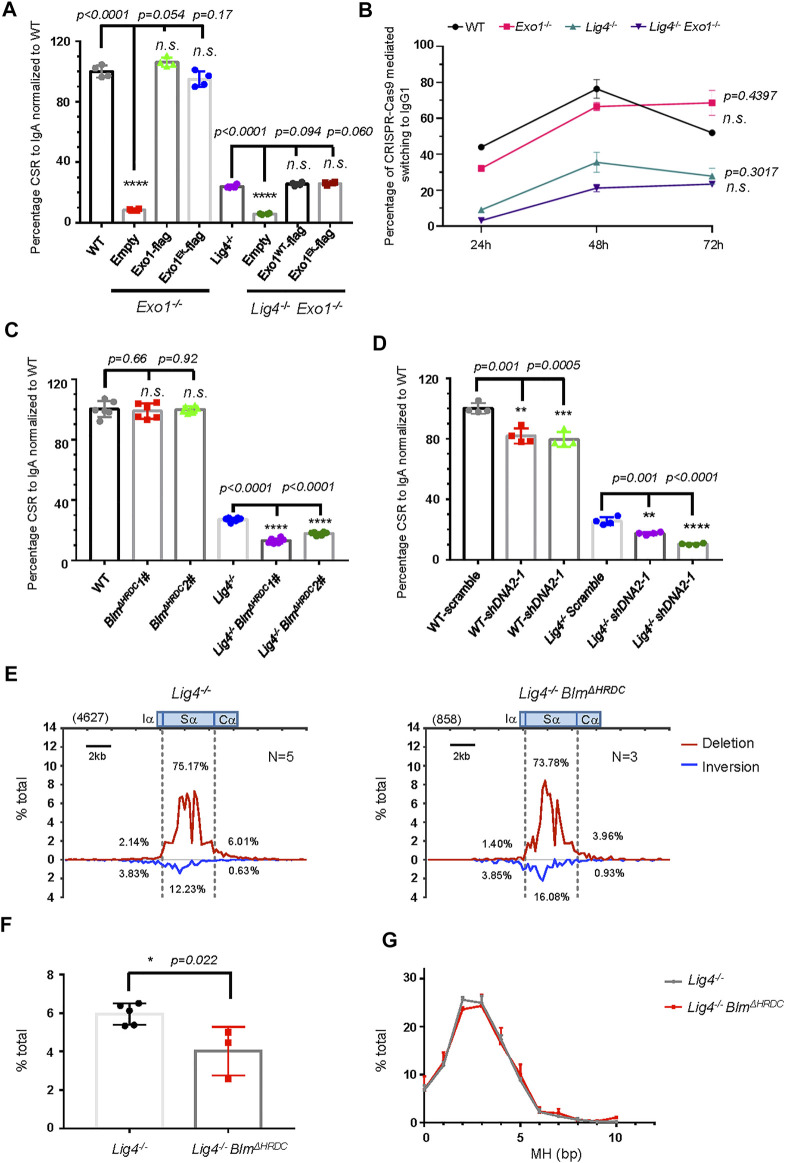
Long resection factors BLM/DNA2 are involved in A-EJ mediated CSR. **(A)** Normalized CSR to lgA in Exo1-deficient B cell reconstituted with retrovirus expressing indicated constructs. Data were presented as mean ± SD from four independent experiments (Student’s *t*-test, *p < 0.05, **p < 0.01, ***p < 0.001, ****p < 0.0001, n. s. (*p* > 0.05) indicates non-significant differences). **(B)** Efficiency of switching to lgG1 with CRISPR/Cas9 targeting Sμ and Sγ1, respectively, in Exo1-deficient WT and *Lig4* ^*−/−*^ cells. Data were presented as mean ± SD from three independent experiments (two-way ANOVA, n. s. (*p* > 0.05) indicates non-significant differences). **(C)** Normalized IgA CSR efficiency in *DNA2*-silenced WT and *Lig4* ^*−/−*^ cells. Data were presented as mean ± SD from four independent experiments (Student’s *t*-test, *p < 0.05, **p < 0.01, ***p < 0.001, ****p < 0.0001, n. s. (*p* > 0.05) indicates non-significant differences). **(D)** Normalized IgA CSR efficiency in *Blm* HRDC domain deleted WT and *Lig4* ^*−/−*^ cells. Data were presented as mean ± SD from six independent experiments (Student’s *t*-test, *p < 0.05, **p < 0.01, ***p < 0.001, ****p < 0.0001, n. s. (*p* > 0.05) indicates non-significant differences). **(E)** Linear distribution of pooled Sμ-Sα junctions recovered from HTGTS libraries with CSR activated *Lig4* ^*−/−*^ *Blm* ^ΔHRDC^ cells. Numbers (*N*) indicated experiment repeats. **(F)** Percentage of long resection junctions in *Lig4* ^*−/−*^ *Blm* ^ΔHRDC^ cells. Data were presented as mean ± SEM (Student’s *t*-test, *p < 0.05, n. s. (*p* > 0.05) indicates non-significant differences). **(G)** The MH pattern of Sμ-Sα junctions in *Lig4* ^*−/−*^ *Blm* ^ΔHRDC^ cells. Data were presented as mean ± SEM.

To study the role of long range DSB resection factors in CSR, we first deleted Exo1 by CRISPR/Cas9 in CH12F3 cells (Supplementary Figures S5A,B). Consistent with previous reports, IgA switching in Exo1 knockout cells in both WT and *Lig4* ^*−/−*^ backgrounds was extremely low (Supplementary Figure S5C) due to severe defect in mismatch repair that is critical to convert AID-initiated lesions into DSBs (Bardwell et al., 2004). Except for its exonuclease activity, Exo1 also has a structural function to facilitate the assembly of high-order protein complex. The Exo1^E109K^ mutation that is exonuclease-dead has been shown to retain mismatch repair activity but is defective in DSB resection and HR-related functions (Schaetzlein et al., 2013). We thus reintroduced the Exo1^E109K^ (referred to as Exo1^EK^ hereafter) mutation by retrovirus back to *Exo1*-deleted WT and *Lig4* ^*−/−*^ cells (Supplementary Figure S5D), and both Exo1^WT^ and Exo1^EK^ fully rescued the near-null IgA switching in not only *Exo1* ^*−/−*^ cells, but also *Lig4* ^*−/−*^ *Exo1* ^*−/−*^ cells (Figure 3A, Supplementary Figure S5E), indicating that exonuclease-embedded DSB resection function of Exo1 is not required for either c-NHEJ or A-EJ-mediated CSR. To further test whether Exo1 plays any role in joining of non-AID initiated DSBs, we introduced simultaneous blunt end breaks at Sμ and Sγ1 by CRISPR/Cas9 (Supplementary Figure S5F) and tested switching to IgG1 in WT, *Lig4* ^*−/−*^, *Exo1* ^*−/−*^, or double-mutant cells at 24, 48, and 72 h post-transfection. After normalization with transfection efficiency, efficient joining of Sμ-Cas9 DSBs to Sγ1-Cas9 breaks generated 40–60% of IgG1+ cells; as expected, Lig4 ablation reduced IgG1 switching efficiency by more than half to only 10–20% (Figure 3B, Supplementary Figures S5G,5H). In addition, we found that *Exo1* deletion in either in WT or *Lig4* ^−/−^ cells did not reduce Cas9-mediated IgG1 switching than the corresponding controls (Figure 3B). Taken together, we conclude that Exo1 is not required for joining AID or Cas9-generated DSBs by either c-NHEJ or A-EJ pathways.

We then asked whether DNA2/BLM-mediated long range resection is required for efficient A-EJ. Two specific shRNA efficiently knocked down the mRNA expression of DNA2 by 50–70% in both wild type and *Lig4* ^*−/−*^ CH12F3 cells (Supplementary Figure S6A); accordingly, the IgA CSR efficiency in WT cells was slightly decreased by about 20%, and *Lig4* ^*−/−*^ cells with shDNA2 exhibited an IgA CSR decline by 30% (Figure 3C, Supplementary Figure S6H). This deficiency can be at least partly attributed to impaired proliferation caused by DNA2 knockdown in WT and *Lig4* ^*−/−*^ cells (Supplementary Figure S6B). To test the role of BLM in A-EJ, we first deleted with CRISPR/Cas9 the exon 8 of *Blm* gene upstream of the helicase domain; this mutation rendered ablation of BLM by premature termination of translation (Babbe et al., 2009) (Supplementary Figures S6C,S6D). The resultant BLM^Δhelicase^ cells were indistinguishable in IgA switching compared with WT cells, whilst BLM^Δhelicase^ in the *Lig4* ^*−/−*^ background slightly but significantly reduced IgA CSR compared to control cells (Supplementary Figure S6E). Due to severe slow proliferation caused by helicase domain disruption, we generated another *Blm* mutation by Cas9 to delete the exon 19 that encodes Helicase-and-RNaseD-like-C-terminal (HRDC) domain of BLM (Supplementary Figures S6F,G). The HRDC domain interacts with the ATPase domain of BLM that may affect its helicase activity (Newman et al., 2015) and has been shown to be required for annealing of complementary single-strand DNA and Holliday junction resolution (Wu et al., 2005; Newman et al., 2015; van Wietmarschen et al., 2018). Although BLM^ΔHRDC^ CH12F3 cells proliferate and switch to IgA normally, BLM^ΔHRDC^ in *Lig4* ^*−/−*^ background displayed a substantial decrease in CSR efficiency (Figure 3D, Supplementary Figures S6l), indicating that the HRDC domain of BLM is required for A-EJ, but not c-NHEJ-mediated class switching.

To gain more insights on the mechanism of how BLM participates in A-EJ, we performed HTGTS assay in WT and *Lig4* ^*−/−*^ cells with BLM^ΔHRDC^ mutation and analyzed the pattern of S-S joining and MH in these cells (Supplementary Figure S7). Compared with WT cells, the BLM^ΔHRDC^ mutant exhibited a slightly decrease in the proportion of Sα junctions and a mild increase in junctions involving long Sα resection (Supplementary Figures S7B,C). When examining MH pattern of Sμ-Sα junctions, we recovered no significant difference between WT and BLM^ΔHRDC^ mutant cells (Supplementary Figure S7D). In contrast, a significant decrease in the proportion of long Sα resection junctions in *Lig4* ^*−/−*^ cells with BLM^ΔHRDC^ mutation was observed (Figures 3E,F), indicating the HRDC activity is required for the joining of long-resected Sα breaks in *Lig4* ^*−/−*^ cells. Similar to aforementioned cells in WT background, no significant difference in the MH profile in Sμ-Sα junctions was observed in BLM^ΔHRDC^ cells compared with the corresponding *Lig4* ^*−/−*^ control (Figure 3G).

### ATM Kinase Activity is Required for Both c-NHEJ and A-EJ-Mediated Class Switch Recombination

**FIGURE 4:**
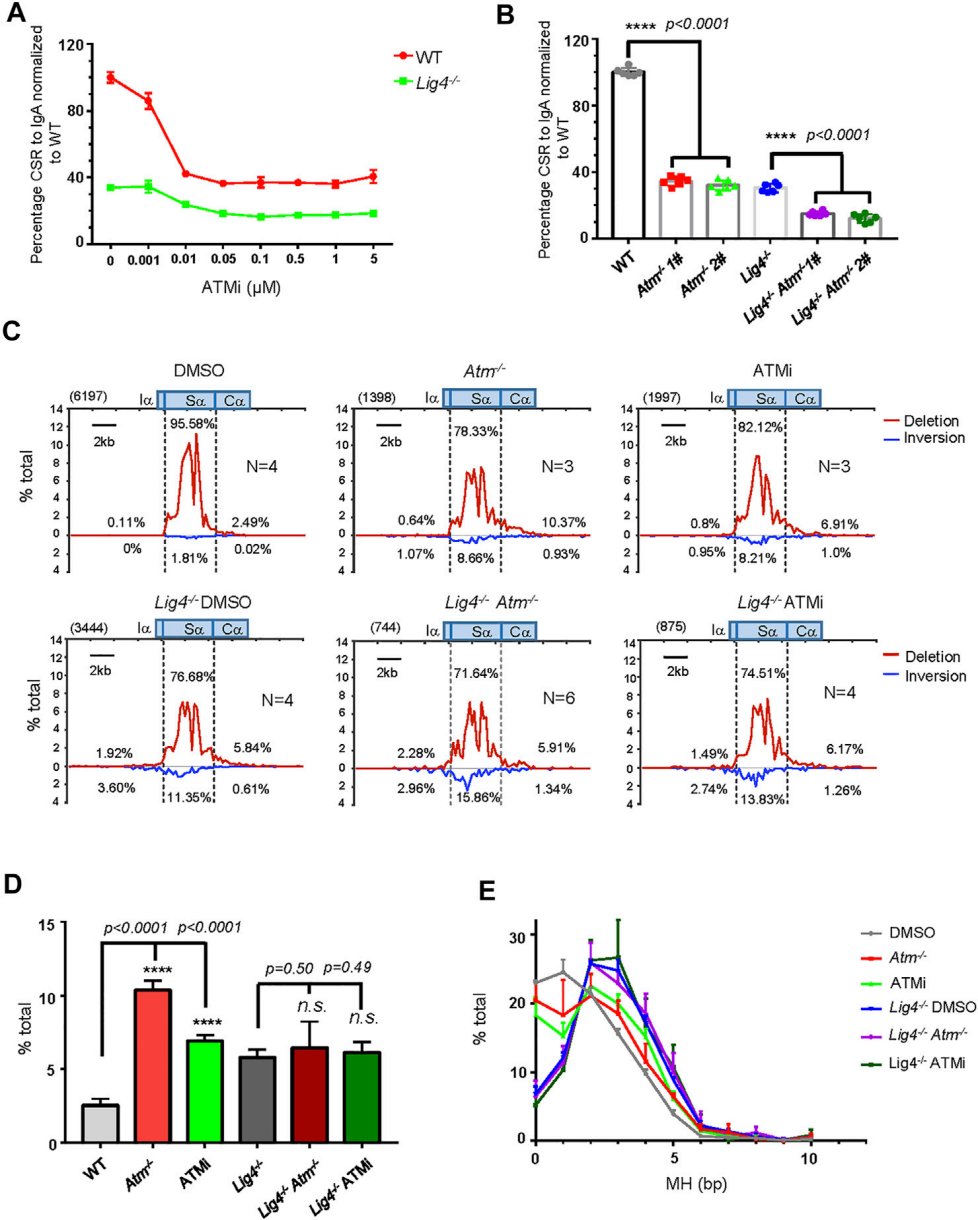
ATM and its kinases activity play important role in A-EJ mediated CSR. **(A)** Normalized lgA switching efficiency in WT and *Lig4* ^*−/−*^ cells treated with ATM inhibitor AZD1390 at gradient concentrations ranging from 0 μM, 0.001 μM, 0.01 μM, 0.05 μM, 0.1 μM, 0.5 μM, and 1–5 μM. Data were presented as mean ± SD from three independent experiments. **(B)** Normalized lgA switching efficiency in *Atm* deleted WT and *Lig4* ^*−/−*^ cells. Data were presented as mean ± SD from six independent experiments (Student’s *t*-test, *p < 0.05, **p < 0.01, ***p < 0.001, ****p < 0.0001, n. s. (*p* > 0.05) indicates non-significant differences). **(C)** Linear distribution of pooled Sμ-Sα junctions recovered from HTGTS libraries with CSR activated DMSO or 0.1 μM AZD1390-treated ATM-deficient WT and *Lig4* ^*−/−*^ cells. Numbers (*N*) indicated experiment repeats. **(D)** Percentage of long resection junctions in ATM inhibitor-treated and ATM-deficient WT and *Lig4* ^*−/−*^ cells. Data were presented as mean ± SEM (Student’s *t*-test, *p < 0.05, **p < 0.01, ***p < 0.001, ****p < 0.0001, n. s. (*p* > 0.05) indicates non-significant differences). **(E)**The usage of MH among Sμ-Sα junctions recovered from HTGTS libraries ATM inhibitor-treated and ATM-deficient WT and *Lig4* ^*−/−*^ cells. Data were presented as mean ± SEM.

AID-initiated DSB recruits and activates ATM. While ATM positively regulates c-NHEJ-mediated CSR, its role in A-EJ has not been carefully examined. To this end, we first applied a highly selective ATM inhibitor AZD1390 that suppressed c-NHEJ mediated CSR at low concentrations with no effect on germline transcription and AID expression (Figure 4A, Supplementary Figures S8C,E). Treating *Lig*4^−/−^ cells with AZD1390 further reduced IgA switching by about half (Figure 4A). The CSR reduction by AZD1390 treatment in *Lig4* ^*−/−*^ cells was phenocopied by *Atm* knockout (Figure 4B, Supplementary Figure S8A). In addition, we observed more phosphorylation of KAP1 that was ATM-dependent in IR-irradiated Lig4-deficient cells compared with WT control, indicating persistent ATM activation in A-EJ cells (Supplementary Figure S8B). Together, we concluded that ATM kinase activity is required for both c-NHEJ and A-EJ-mediated CSR.

We then examined the Sμ-Sα joining pattern with ATM deletion and ATM kinase inhibition by the HTGTS assay. *Atm* knockout or kinase activity inhibition in CH12F3 cells similarly resulted in increased Sα DSBs resection, increased MH usage, and decreased ratio of deletional versus inversional Sμ-Sα joining (Figures 4C–E). Surprisingly, in *Lig4* ^−/−^ cells with *Atm* ablation, although the percentage of Sα deletional joining over total *IgH* junctions decreased (Supplementary Figures S8F-H) that was consistent with further reduced IgA surface expression than *Lig4* ^*−/−*^ cells by flow cytometry, other parameters including Sα DSBs long resection, MH usage, and ratio of deletional versus inversional Sμ-Sα junctions remained unchanged compared with controls (Figures 4C–E). We concluded that while ATM plays important roles in both c-NHEJ and A-EJ-mediated CSR, it did not appear to control Sα long resection in Lig4-deficient cells. We also examine the potential role of another PIKK, DNA-PKcs in CSR. Consistent with the reported role of DNA-PKcs in promoting c-NHEJ to IgG in primary mouse B cells (Franco et al., 2008; Callén et al., 2009), while deleting DNA-PKcs by CRISPR/Cas9 (Supplementary Figures S9A–D) significantly diminished IgA in WT CH12F3 cells, knocking out DNA-PKcs in *Lig4* ^*−/−*^ cells did not further reduce IgA CSR (Supplementary Figure S9E), indicating that DNA-PKcs does not play a role in A-EJ.

### ATM Functions Independently of Mre11/CtIP in Promoting A-EJ

**FIGURE 5:**
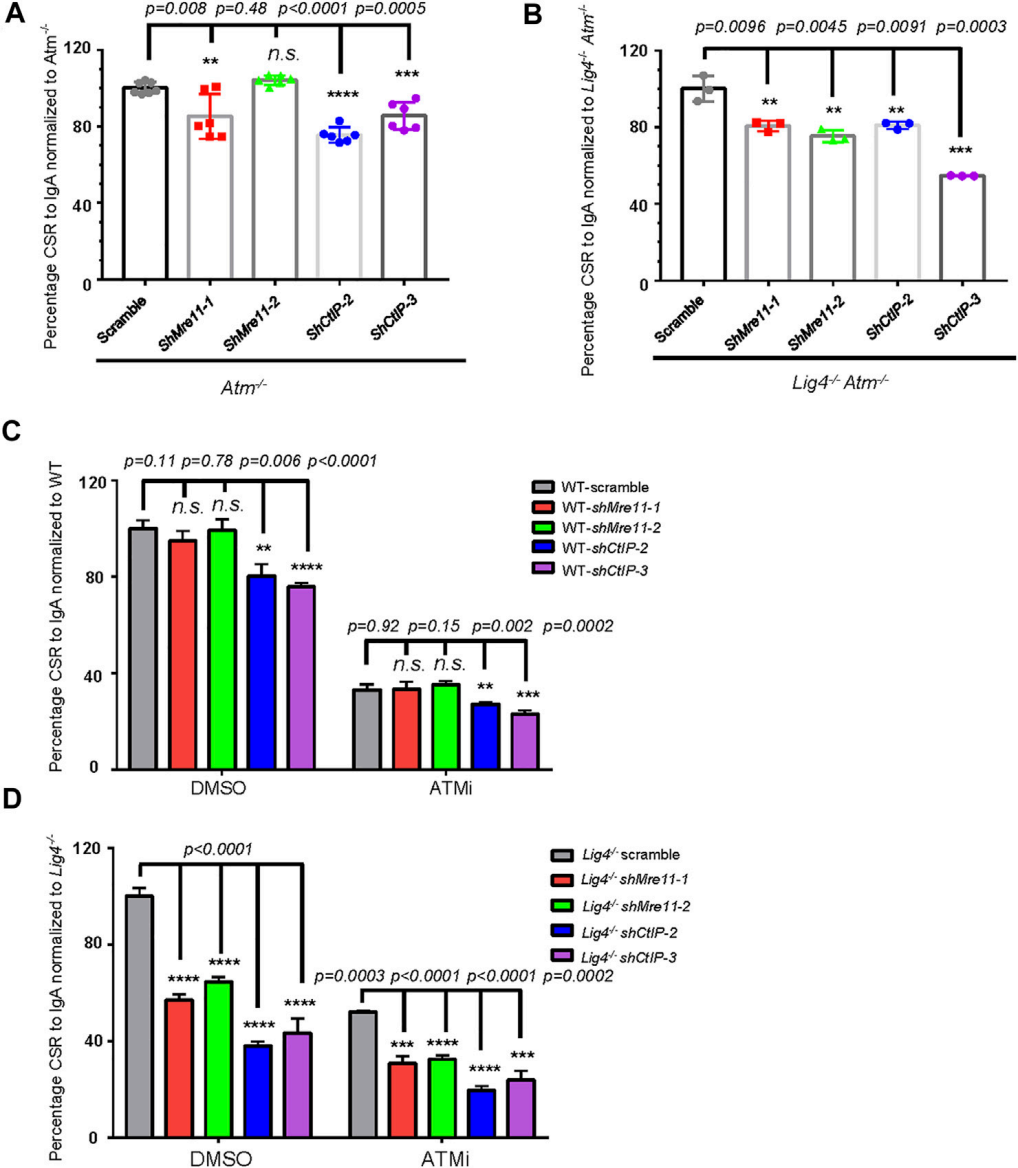
ATM and Mre11/CtIP promote A-EJ mediated CSR independently of each other. **(A, B)** Silencing Mre11/CtIP by different shRNAs further reduced CSR in both *Atm* ^*−/−*^ cells (**A**) and *Lig4* ^*−/−*^ *Atm* ^*−/−*^ cells (**B**). Data were presented as mean ± SD from six in **A**, three in **B** independent experiments (Student’s *t*-test, *p < 0.05, **p < 0.01, ***p < 0.001, ****p < 0.0001, n. s. (*p* > 0.05) indicates non-significant differences). **(C)** Inhibition of ATM kinase activity with AZD1390 (100 nM) could reduce CSR in Mre11/CtIP-silenced WT CH12F3 cells. Data were presented as mean ± SD from three independent experiments (Student’s *t*-test, *p < 0.05, **p < 0.01, ***p < 0.001, ****p < 0.0001, n. s. (*p* > 0.05) indicates non-significant differences). **(D)** Inhibition of ATM kinase activity with AZD1390 could further reduce IgA CSR in Mre11/CtIP-silenced *Lig4* ^*−/−*^ cells. Data were presented as mean ± SD from three independent experiments (Student’s *t*-test, *p < 0.05, **p < 0.01, ***p < 0.001, ****p < 0.0001, n. s. (*p* > 0.05) indicates non-significant differences).

Previous reports suggested ATM may promote DSBs resection through phosphorylating CtIP as the CtIP-T859A mutant compromised resection and HR repair (Peterson et al., 2013; Wang et al., 2013). To investigate the relationship between ATM and Mre11/CtIP in end joining during CSR, we first silenced Mre11 or CtIP expression by shRNA in *ATM* ^*−/−*^ cells (Supplementary Figure S10A). While shMre11 in *ATM* ^−/-^ cells showed only a mild effect on IgA + cells by surface staining, silencing CtIP slightly decreased (∼20%) IgA switching compared with scramble control (Figure 5A). However, in *Lig4* ^*−/−*^ *ATM* ^*−/−*^ cells, silencing either Mre11 or CtIP (Supplementary Figure S10B) conferred significantly more defect in CSR up to 50% lower than scramble control, a phenotype much more severe than that in *Atm* deletion alone (Figure 5B), suggesting that Mre11 and CtIP function in part via different pathways than ATM in promoting A-EJ in Lig4-deficient cells.

To further confirm the above observation, we treated Mre11 or CtIP-silenced WT or *Lig4* ^*−/−*^ cells with DMSO or AZD1390 before stimulating them for CSR. Consistent with aforementioned findings, silencing only CtIP but not Mre11 in DMSO-treated WT cells resulted in a mild IgA switching defect (Figure 5C). As expected, ATMi treatment decreased IgA switching of scramble control cells compared with DMSO treatment; no significant difference in switch efficiency was observed between ATMi-treated control and shMre11 cells (Figure 5C). In contrast, ATMi-treated shCtIP cells showed further IgA defect than scramble control cells under the same treatment (Figure 5C). These data confirmed an epistatic effect between ATM and Mre11, and a non-overlapping function of ATM and CtIP in c-NHEJ-mediated cells. In stark contrast, ATMi treatment indeed caused more severe switching defect in *Lig4* ^*−/−*^ cells infected with either shMre11 or shCtIP compared with that of scramble control (Figure 5D). Taken together, these data implicated that while ATM and Mre11 are epistatic in c-NHEJ-mediated CSR, it functions at least in part independent of Mre11 and CtIP in promoting A-EJ-mediated CSR in *Lig4* ^*−/−*^ cells.

### Less Dependency on Mre11/CtIP-Mediated Resection for Class Switch Recombination in *53bp1* ^−/−^ Cells

**FIGURE 6:**
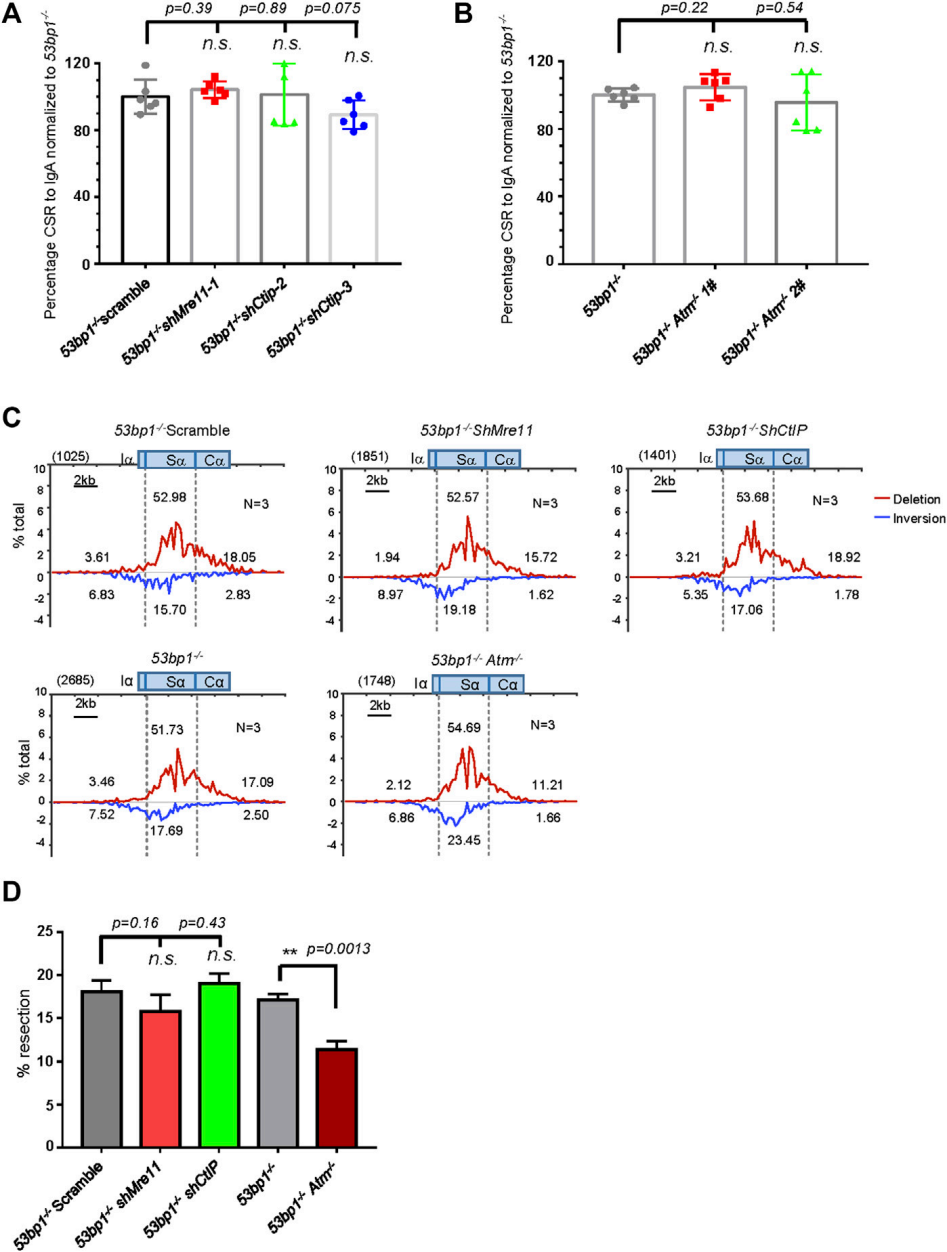
Mre11/CtIP and ATM are not required for CSR in 53bp1^−/−^ cells. **(A)** Silencing Mre11/CtIP did not affect IgA CSR in *53bp1* ^*−/−*^ cells. Data were presented as mean ± SD from six independent experiments (Student’s *t*-test, n. s. (*p* > 0.05) indicates non-significant differences). **(B)** Normalized lgA switching efficiency in *Atm*-deleted *53bp1* ^*−/−*^ cells. Data were presented as mean ± SD from six independent experiments (Student’s *t*-test, n. s. (*p* > 0.05) indicates non-significant differences). **(C)** Linear distribution of pooled Sμ-Sα junctions recovered from HTGTS libraries with CSR activated Mre11/CtIP-silenced and ATM knockout *53bp1* ^*−/−*^ cells. Numbers (*N*) indicated experiment repeats. **(D)** Percentage of long resection junction in Sμ-Sα junctions recovered from HTGTS libraries with Mre11/CtIP-silenced and ATM-deficient *53bp1* ^*−/−*^ cells. CSR and HTGTS analysis were assayed at 72 h after stimulation with α-CD40/IL-4/TGF-β. Data were presented as mean ± SEM (Student’s *t*-test, *p < 0.05, **p < 0.01, n. s. (*p* > 0.05) indicates non-significant differences).

Ours and others’ previous reports had indicated that 53BP1-deficient B cells underwent greatly impaired CSR characterized by significantly increased S region DSB resection and MH usage in S-S junctions (Bothmer et al., 2013; Dong et al., 2015), representing a scenario similar to A-EJ comparing to c-NHEJ factor ablation. To investigate whether Mre11/CtIP-mediated resection plays any role in A-EJ in 53BP1-deficient cells, we first silenced these two factors with lentiviral expressed shRNA (Supplementary Figure S11A) and stimulated them for switching. However, in contrast to what we observed in *Lig4* ^*−/−*^ cells, silencing Mre11 or CtIP in *53bp1* ^*−/−*^ cells did not lead to further defect in IgA switching compared with that of scramble control (Figure 6A). ATM kinase inhibition by AZD1390 in *53bp1* ^*−/−*^ cells resulted in very mild, if there was any, additive decline in switching than DMSO controls (Supplementary Figure S11B). Similarly, *Atm* gene knockout by CRISPR/Cas9 in *53bp1* ^*−/−*^ cells (Supplementary Figures S11C,D) did not change its switching efficiency (Figure 6B), suggesting that ATM and its kinase activity are not required for A-EJ in this setting.

We also deleted resection factor EXD2 in *53bp1* ^*−/−*^ cells and results showed EXD2 depletion did not further reduce IgA switching (Supplementary Figures S11E,F). Similar to the observations with WT CH12F3 cells, deleting Exo1 in *53bp1* ^*−/−*^ nearly completely abolished switching, and re-introducing either EXO1^WT^ or Exo1^E109K^ mutants back to the *53bp1* ^*−/−*^ *Exo1* ^*−/−*^ double-mutant cells restored IgA switching to the *53bp1* ^−/−^ level (Supplementary Figures S11G–I), suggesting that Exo1-mediated DSB resection was not required for A-EJ in 53BP1-deficient cells. Taken together, it appears that neither short-range nor long-range resections factors were critical for A-EJ in 53BP1-deficient cells, and the requirement for resection activity in A-EJ-mediated CSR is context dependent.

To gain more insight into the effect of Mre11/CtIP and ATM kinase on class switch in *53bp1* ^*−/−*^ cells, we applied HTGTS assay with Mre11/CtIP-silenced or *Atm*-deleted *53bp1* ^*−/−*^ cells. Consistent with the IgA FACS staining data, HTGTS revealed similar levels of Sμ-Sα joining in all cells with shMre11 or shCtIP or *ATM* deletion compared with *53bp1* ^*−/−*^ control cells (Supplementary Figures S12A,B). Surprisingly, we found that Mre11 or CtIP silencing did not change the overall pattern of Sα joining in *53bp1* ^*−/−*^ cells in that deletional versus inversional Sα joins and levels of SαDSB resection were similar in shMre11 and shCtIP-infected *53bp1* ^*−/−*^ cells compared with those in scramble control cells (Figures 6C,D, Supplementary Figure S12C). However, we indeed observed a significant increase in the percentage of direct junctions and junctions with 1 base pair of microhomology sequences in shMre11 cells (Supplementary Figure S12D), implicating that Mre11 and CtIP may have different roles in mediating MH-mediated joining in this context. *Atm* gene deletion, on the other hand, resulted in significantly decreased Sα DSB long resection without changing MH usage in Sα junctions (Figures 6C,D, Supplementary Figure S12D). Taken together, we concluded that while only ATM activity is required for Sα DSB resection in 53BP1-deficient cells, neither Mre11/CtIP nor ATM is required for the residual switching in this setting.

## Discussion

Our previous studies reported elevated S region DSB resection and MH usage in 53*bp1* ^−/−^ or *Lig4* ^−/−^ than that in wild type cells (Dong et al., 2015; Panchakshari et al., 2018). In this study, we continue to demonstrate that DSB resection factors together with ATM play important roles in meditating A-EJ in the absence of Lig4. Based on the findings from this study and others’ reports, we summarize the roles of each individual factor in CSR in the absence of Lig4 or 53BP1. Consistent with a prior report (Dinkelmann et al., 2009), the mild increase in resection and MH usage in Mre11-silenced cells that resembled (but not as severe) ATM-deficient cells supports the notion that Mre11 has a minor role in meditating c-NHEJ in wild type B cells through activating ATM-dependent DDR. In contrast, CtIP deficiency impaired CSR but the nearly identical resection and junctional MH as control cells indicates that CtIP is not strictly required for end joining in WT cells. The mild CSR defect in CtIP-deficient WT cells had been attributed to impaired cellular proliferation or AID expression (Lee-Theilen et al., 2011; Liu et al., 2019). Our data that several shCtIP clones with different proliferation rates and AID levels showed similar efficiency indicated other mechanisms may underlie CtIP’s role in CSR. An interesting hypothesis is that CtIP facilitates DSB end bridging independent of resection initiation, as a recent study suggested (Öz et al., 2020), likely through multimerization (Wang et al., 2012; Andres et al., 2015; Davies et al., 2015). Furthermore, our data clearly supported roles for Mre11/CtIP in A-EJ-mediated CSR in *Lig4* ^*−/−*^ cells. However, it appeared that Mre11/CtIP silencing did not affect S region DSB long resection indicated by the distal Cα junctions in HTGTS assay. As AID-initiated Sα breaks are highly enriched in the core, joining of Sα breaks into Cα would require an Sα DSBs to be resected over a thousand base pairs away from the core region. Thus, the lack of change in Cα junctions in shMre11/shCtIP cells can be explained as that they do not affect Sα DSB “long” resection, and their potential roles in mediating Sα short resection in Lig4-deficient cells cannot be ruled out. In addition, CtIP may have additional Mre11-independent roles in A-EJ in *Lig4* ^*−/−*^ cells in the same fashion in WT cells. In this regard, we indeed found that shCtIP *Lig4* ^*−/−*^ cells showed further decreased ratio of Sα direct versus inversional junctions that was not seen in shMre11 cells, indicating CtIP can participate in A-EJ by promoting deletional Sμ-Sα joining independent of Mre11.

As Mre11 or CtIP silencing further reduced but not completely abolished CSR, there exists Mre11/CtIP-independent A-EJ activities in Lig4-deficient cells that include BLM/DNA2-mediated long range resection, as deleting *Blm* or silencing DNA2 in *Lig4* ^*−/−*^ cells reduced residual switching. BLM may participate in A-EJ-mediated CSR through two mechanisms. First, it contributes to S region DSB resection in *Lig4* ^*−/−*^ cells by using its helicase activity and in conjunction with DNA2. However, the severe proliferation defect due to impaired S phase DSB repair in Lig4-deficient CSR-activated cells hindered further investigation on this function. Second, BLM may promote resection-generated ssDNA to anneal with each other by its HRDC domain (Wu et al., 2005). We cannot prove or exclude at this point the possibility that the HRDC domain may affect the helicase activity through intramolecular interaction (Newman et al., 2015). To our surprise, the DSB resection activity of Exo1 is not strictly required for A-EJ in this context, an observation consistent with a prior report that the separation-of-function mutation Exo1^E109K^ can still support A-EJ activity in an I-SceI-based assay system (Schaetzlein et al., 2013). This finding was further confirmed by a CRISPR/Cas9-mediated end joining assay that does not rely on Exo1 to generate S region DSBs, supporting the notion that Exo1 and associated resection activity is not required for A-EJ in general.

Corresponding to a recent report (Wang et al., 2020), our data demonstrated that ATM and its intrinsic kinase activity were required for both c-NHEJ and A-EJ-mediated CSR, and there were no obvious differences between ATM kinase inhibition and *Atm* deletion in A-EJ efficiency in Lig4-deficient cells. In c-NHEJ, ATM functions in part to recruit 53BP1-Rif1 to prevent BRCA1/CtIP-mediated S region DSB resection. However, we did not find significant changes in Sα DSBs resection and MH usage in *Atm* deleted or kinase inhibited *Lig4* ^*−/−*^ cells. Prior reports have suggested that ATM promotes CtIP-dependent DSB resection by direct phosphorylation (Wang et al., 2020), our finding with additive CSR impairment in Lig4-deficient cells defective with both ATM and CtIP clearly indicated that ATM activates additional targets in A-EJ other than CtIP.

Lastly, our data revealed the differential needs for Mre11/CtIP/ATM between Lig4-deficient and 53BP1-deficient cells that could stem from the readiness of these cells to undergo resection. In Lig4-deficient cells, 53BP1 is still present at DSBs to recruit Rif1 to counteract CtIP-mediated resection in G1, and a fraction of cells may proceed to S/G2 phase for CtIP/BRCA1 to block Rif1 recruitment to enable resection (Daley and Sung, 2013; Di Virgilio et al., 2013). It is thus conceivable that silencing Mre11 or CtIP in *Lig4* ^*−/−*^ cells can lead to resection inhibition and dampen A-EJ efficiency. On the other hand, in *53bp1* ^*−/−*^ cells the requirement for CtIP to exclude 53BP1/Rif1 becomes minimal as resection suppression by Rif1 has been canceled out. Consistent with the minimal effect of resection on CSR efficiency we observed in *53bp1* ^*−/−*^ cells, a recent report revealed that resection can be largely uncoupled with CSR (Sundaravinayagam et al., 2019) and suggested that higher order chromatin structure by 53BP1 oligomerization is essential to enforce the 3-D architecture of *IgH* locus for efficient class switching (Dong et al., 2015; Fernandez and Chaudhuri, 2019). In this context, resection inhibition in *53bp1* ^*−/−*^ cells by either Mre11/CtIP silencing or ATM ablation did not change A-EJ efficiency, as the requisite *IgH* loops for efficient CSR has already greatly collapsed (Wuerffel et al., 2007; Feldman et al., 2017; Zhang et al., 2019) (Cortizas et al., 2013).

## Data Availability

The datasets presented in this study can be found in online repositories. The names of the repository/repositories and accession number(s) can be found in the article/Supplementary Material.
