# Supplementary material for: Multiple DSB Resection Activities Redundantly Promote Alternative End Joining-Mediated Class Switch Recombination
Source: Front Cell Dev Biol. 2021 Nov 26;9:767624. doi: 10.3389/fcell.2021.767624 (PMC8671047; doi:10.3389/fcell.2021.767624)
Supplement: Supplementary file 1 [file DataSheet2.PDF]

Table S1. Oligonucleotides

Name	Sequence 5’->3’	Sourse
EXD2-5’gRNA	GAATACCCCATATTCCATGC	This paper
EXD2-3’gRNA	GTTATAAACCAGTGACCCAC	This paper
EXD2-Detect -Forward	GTTGGTAAGCTGTGTCACCT	This paper
EXD2-Detect -Reverse	ACTCCCACACAGTCACTCAC	This paper
EXO1-5’gRNA	GTCCTCTGAGTAGTCACAGT	This paper
EXO1-3’gRNA	TCCACGTGATAAGGAAGCAT	This paper
EXO1-Detect -Forward	TCAAATCCCTTGGGTGCGAA	This paper
EXO1-Detect -Reverse	GCACGGATAAACCCTGGTCA	This paper
ATM-5’gRNA	GTCCTCAGTCGATTATCACT	Panchakshari et al., 2018
ATM-3’gRNA	TATCTTGATAAACGAGCAGT	Panchakshari et al., 2018
ATM-Detect -Forward	CACAGATGCCCTTATATCCTGC	This paper
ATM-Detect -Reverse	CTCTCAACTTCCTGGTCCAAATC	This paper
DNA-PKcs-5’gRNA	GCCTCACATAAGCTAAGTCA	This paper
DNA-PKcs-3’gRNA	GCTGATGCACAGCAGTGCAT	This paper
DNA-PKcs-Detect -Forward	TTCTTGTACCTGACTCTGTTG	This paper
DNA-PKcs-Detect -Reverse	GGTTGTCAACTTCCTCTGGAATCA	This paper
Blm$^{\Delta HRDC}$-5’gRNA	GTATGTGTGTCATGGTGTAG	This paper
Blm$^{\Delta HRDC}$-3’gRNA	GTGGCCAGTTCATCTACTAA	This paper
Blm$^{\Delta HRDC}$-Detect -Forward	TGAATGACGCTGAGCCTGTA	This paper
Blm$^{\Delta HRDC}$-Detect -Reverse	GGAAAGCAAGGCCTGAACAA	This paper
Blm$^{\Delta Helicase}$-5’gRNA	GCCAAAGTTGAGAGCAGAAG	This paper
Blm$^{\Delta Helicase}$-3’gRNA	GGAGCTACACTGGAACTCCA	This paper
Blm$^{\Delta Helicase}$-Detect -Forward	TGTTCTGAGGAGCGGGCTTAG	This paper
Blm$^{\Delta Helicase}$-Detect -Reverse	CTGAACCTGCTTCCTAGCCTAACTG	This paper
EXD2-5’gRNA	GAATACCCCATATTCCATGC	This paper
EXD2-3’gRNA	GTTATAAACCAGTGACCCAC	This paper
EXD2-Detect -Forward	GTTGGTAAGCTGTGTCACCT	This paper
EXD2-Detect -Reverse	ACTCCCACACAGTCACTCAC	This paper
EXO1-5’gRNA	GTCCTCTGAGTAGTCACAGT	This paper
EXO1-3’gRNA	TCCACGTGATAAGGAAGCAT	This paper
EXO1-Detect -Forward	TCAAATCCCTTGGGTGCGAA	This paper
EXO1-Detect -Reverse	GCACGGATAAACCCTGGTCA	This paper
ATM-5’gRNA	GTCCTCAGTCGATTATCACT	Panchakshari et al., 2018
ATM-3’gRNA	TATCTTGATAAACGAGCAGT	Panchakshari et al., 2018
ATM-Detect -Forward	CACAGATGCCCTTATATCCTGC	This paper
ATM-Detect -Reverse	CTCTCAACTTCCTGGTCCAAATC	This paper
DNA-PKcs-5’gRNA	GCCTCACATAAGCTAAGTCA	This paper
DNA-PKcs-3’gRNA	GCTGATGCACAGCAGTGCAT	This paper
DNA-PKcs-Detect -Forward	TTCTTGTACCTGACTCTGTTG	This paper
DNA-PKcs-Detect -Reverse	GGTTGTCAACTTCCTCTGGAATCA	This paper
Blm$^{\Delta HRDC}$-5’gRNA	GTATGTGTGTCATGGTGTAG	This paper
Blm$^{\Delta HRDC}$-3’gRNA	GTGGCCAGTTCATCTACTAA	This paper

Sμ-gRNA	TGGGGTGAGCTGAGCTGAGC	This paper
Sγ1-gRNA	AGCCAGGACAGGTGGAAGTG	This paper
Mre11-shRNA-1-F	GTTAGAGGAAATGATACATTT	Sigma-Aldrich
Mre11-shRNA-2-F	GTAGGCTTGCTGCGCATTAAA	Sigma-Aldrich
CTIP-shRNA-1-F	GCACAGAGACTAGAAGAATTC	This paper
CTIP-shRNA-2-F	ACTTAAGCAAGCCACTATTTA	Sigma-Aldrich
CTIP-shRNA-3-F	GCATTAACCGGCTACGAAAGA	This paper
DNA2-shRNA-1-F	GCTGGCGCACTTGAAGTATTT	This paper
DNA2-shRNA-2-F	GCTGGAGTCGCAATCTAAAGA	This paper
AID-Forward	GAAAGTCACGCTGGAGACCG	Bothmer et al., 2012
AID-Reverse	TCTCATGCCGTCCCTTGG	Bothmer et al., 2012
Hprt-Forward	CACAGGACTAGAACACCTGC	Schuh et al., 1999
Hprt-Reverse	GCTGGTGAAAAGGACCTCT	Schuh et al., 1999
Imμ-Forward	CTCTGGCCCTGCTTATTGTTG	Muramatsu et al., 2010
Cmμ-Reverse	GAAGACATTTGGGAAGGACTGACT	Muramatsu et al., 2010
IαCα-Forward	CCTATGAAGGACACTCAACAACATTG	Han et al., 2011
IαCα-Reverse	ACAGAGCTCGTGGGAGTGTCA	Han et al., 2011
Sμ-gRNA	TGGGGTGAGCTGAGCTGAGC	This paper
Sγ1-gRNA	AGCCAGGACAGGTGGAAGTG	This paper
Mre11-shRNA-1-F	GTTAGAGGAAATGATACATTT	Sigma-Aldrich
Mre11-shRNA-2-F	GTAGGCTTGCTGCGCATTAAA	Sigma-Aldrich
CTIP-shRNA-1-F	GCACAGAGACTAGAAGAATTC	This paper
CTIP-shRNA-2-F	ACTTAAGCAAGCCACTATTTA	Sigma-Aldrich
CTIP-shRNA-3-F	GCATTAACCGGCTACGAAAGA	This paper
DNA2-shRNA-1-F	GCTGGCGCACTTGAAGTATTT	This paper
DNA2-shRNA-2-F	GCTGGAGTCGCAATCTAAAGA	This paper
AID-Forward	GAAAGTCACGCTGGAGACCG	Bothmer et al., 2012
AID-Reverse	TCTCATGCCGTCCCTTGG	Bothmer et al., 2012
Hprt-Forward	CACAGGACTAGAACACCTGC	Schuh et al., 1999
Hprt-Reverse	GCTGGTGAAAAGGACCTCT	Schuh et al., 1999
Imμ-Forward	CTCTGGCCCTGCTTATTGTTG	Muramatsu et al., 2010
Cmμ-Reverse	GAAGACATTTGGGAAGGACTGACT	Muramatsu et al., 2010
IαCα-Forward	CCTATGAAGGACACTCAACAACATTG	Han et al., 2011
IαCα-Reverse	ACAGAGCTCGTGGGAGTGTCA	Han et al., 2011
