# Supplementary material for: Multiple DSB Resection Activities Redundantly Promote Alternative End Joining-Mediated Class Switch Recombination
Source: Front Cell Dev Biol. 2021 Nov 26;9:767624. doi: 10.3389/fcell.2021.767624 (PMC8671047; doi:10.3389/fcell.2021.767624)
Supplement: Supplementary file 2 [file DataSheet1.pdf]

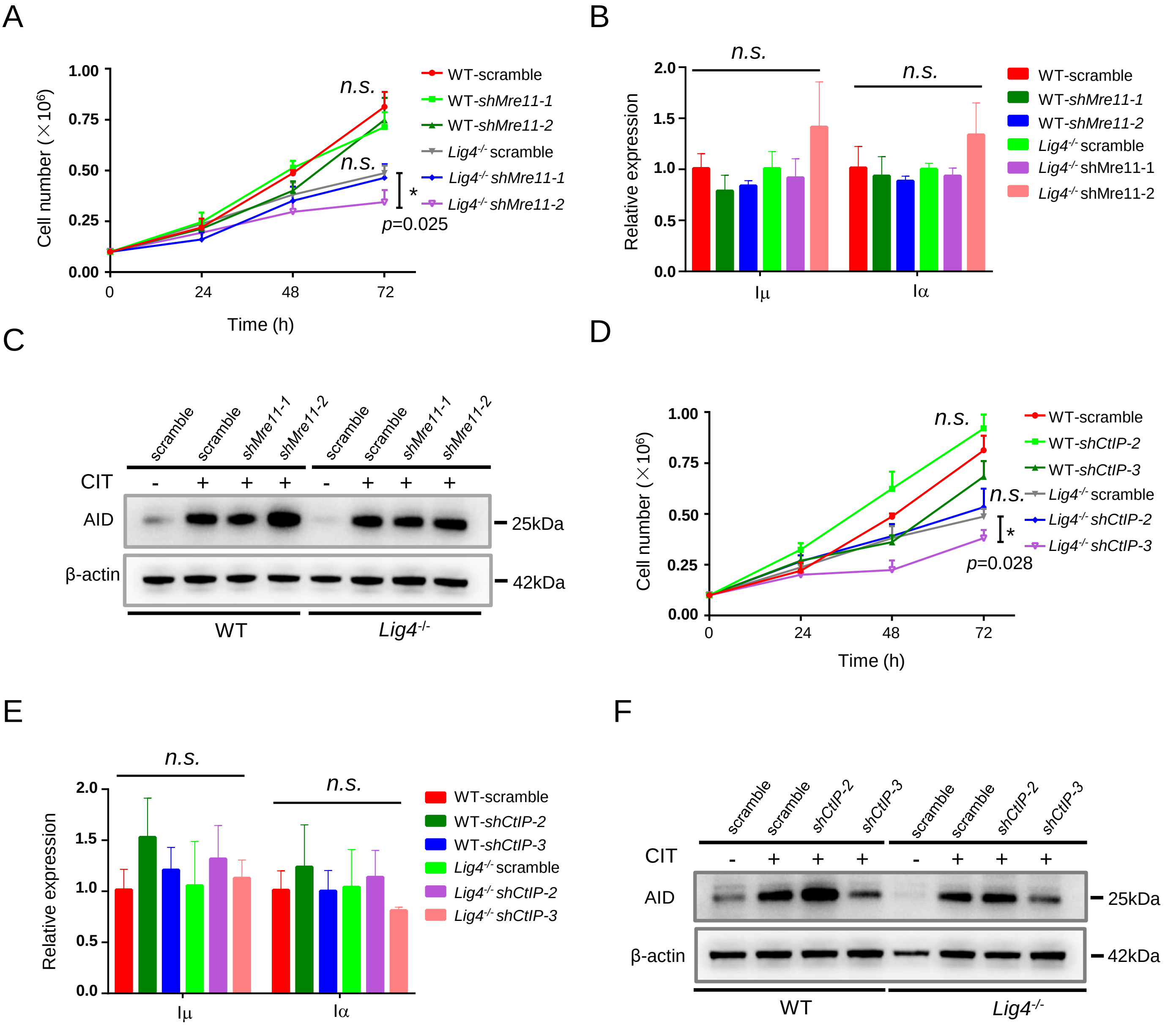

A
n.s.
n.s.
*
p=0.025
Cell number (×10^6)
Time (h)
1.00
0.75
0.50
0.25
0.00
0
24
48
72
WT-scramble
WT-shMre11-1
WT-shMre11-2
Lig4-/- scramble
Lig4-/- shMre11-1
Lig4-/- shMre11-2
B
n.s.
n.s.
Relative expression
2.0
1.5
1.0
0.5
0.0
Iμ
Iα
WT-scramble
WT-shMre11-1
WT-shMre11-2
Lig4-/- scramble
Lig4-/- shMre11-1
Lig4-/- shMre11-2
C
scramble
scramble
shMre11-1
shMre11-2
scramble
scramble
shMre11-1
shMre11-2
CIT
- + + + - + + +
AID
25kDa
β-actin
42kDa
WT
Lig4-/-
D
n.s.
n.s.
*
p=0.028
Cell number (×10^6)
Time (h)
1.00
0.75
0.50
0.25
0.00
0
24
48
72
WT-scramble
WT-shCtIP-2
WT-shCtIP-3
Lig4-/- scramble
Lig4-/- shCtIP-2
Lig4-/- shCtIP-3
E
n.s.
n.s.
Relative expression
2.0
1.5
1.0
0.5
0.0
Iμ
Iα
WT-scramble
WT-shCtIP-2
WT-shCtIP-3
Lig4-/- scramble
Lig4-/- shCtIP-2
Lig4-/- shCtIP-3
F
scramble
scramble
shCtIP-2
shCtIP-3
scramble
scramble
shCtIP-2
shCtIP-3
CIT
- + + + - + + +
AID
25kDa
β-actin
42kDa
WT
Lig4-/-

Supplementary Figure S1. Cell proliferation, germline transcripts and AID expression in activated Mre11/CtIP silenced WT and *Lig4*$^{-/-}$ cells

(A) The proliferation curves of WT and *Lig4*$^{-/-}$ cells transduced with shScramble or shMre11 lentivirus stimulated with α-CD40/IL-4/TGF-β. The cells were collected at 0, 24h, 48h and 72h after stimulation, stained with trypan blue and counted with hemacytometer. Data were presented as mean ± SD from three independent experiments (Student's t-test, $*p < 0.05$, n.s. ($p>0.05$) indicates non-significant differences).

(B) qRT-PCR analysis of spliced mature Iμ-Cμ and Ia-Ca germline transcripts in Mre11-silenced WT and *Lig4*$^{-/-}$ cells. Data were presented as mean ± SD from three independent experiments (Student's t-test, n.s. ($p>0.05$) indicates non-significant differences).

(C) Western blot analysis of AID expression in Mre11-silenced WT and *Lig4*$^{-/-}$ cells.

(D) The proliferation curves of WT and *Lig4*$^{-/-}$ cells transduced with shScramble or shCtIP lentivirus stimulated with α-CD40/IL-4/TGF-β. The cells were collected at 0, 24h, 48h and 72h after stimulation were stained with trypan blue and counted with hemacytometer. Data were presented as mean ± SD from three independent experiments (Student's t-test, $*p < 0.05$, n.s. ($p>0.05$) indicates non-significant differences).

(E) qRT-PCR analysis of spliced mature Iμ-Cμ and Ia-Ca germline transcripts in CtIP-silenced WT and *Lig4*$^{-/-}$ cells. qRT-PCR analysis was performed at 16h after stimulation with α-CD40/IL-4/TGF-β. Data were presented as mean ± SD from three independent experiments (Student's t-test, n.s. ($p>0.05$) indicates non-significant differences).

(F) Western blot analysis of AID expression in CtIP-silenced WT and *Lig4*$^{-/-}$ cells. AID expression was performed at 16h after stimulation with α-CD40/IL-4/TGF-β.

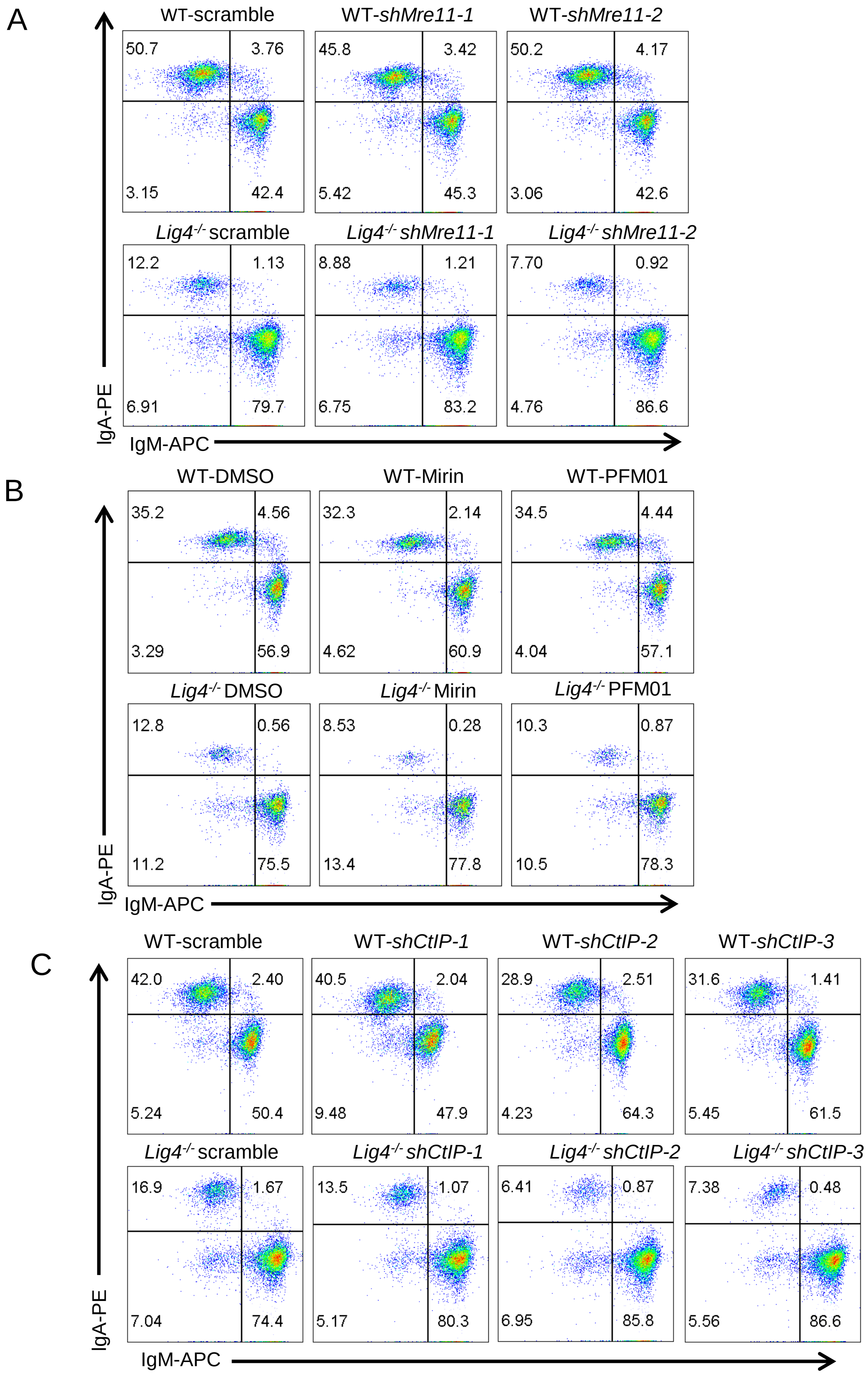

Supplementary Figure S2. Reduced IgA switching in Mre11/CtIP-silenced WT and *Lig4*$^{-/-}$ cells

(A)Representative flow cytometry analysis of switching to lgA in WT and *Lig4*$^{-/-}$ cells transduced with two different shRNAs to silence Mre11 expression.

(B) Representative flow cytometry analysis of switching to lgA in WT and *Lig4*$^{-/-}$ cells treated with Mre11 exonuclease inhibitor (Mirin) and Mre11 endonuclease inhibitor (PFM01).

(C) Representative flow cytometry analysis of switching to lgA in WT and *Lig4*$^{-/-}$ cells transduced with three different shRNAs to silence CtIP expression.

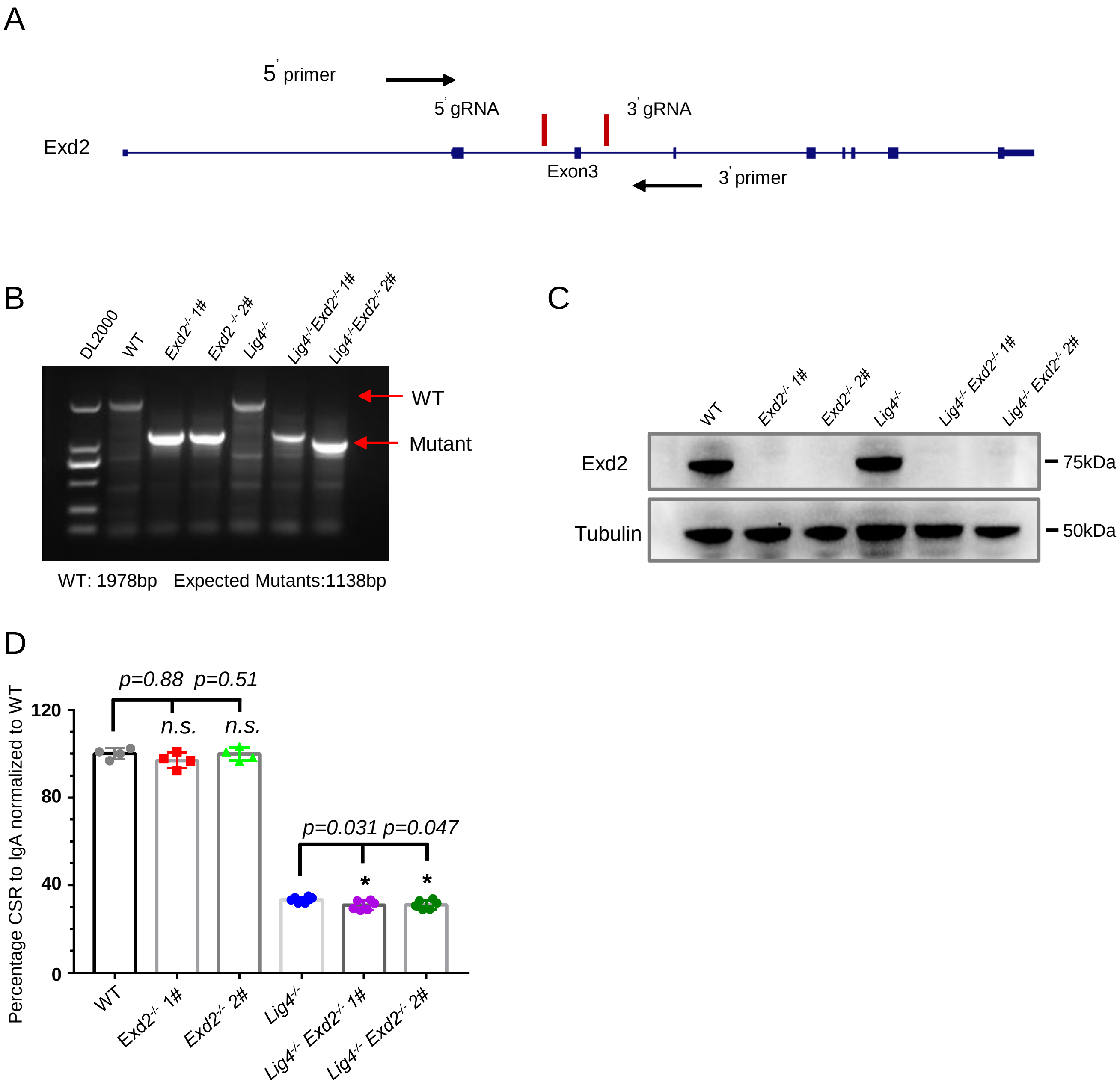

Supplementary Figure S3. Exd2 plays a mild role in A-EJ but not c-NHEJ-mediated CSR
(A)The scheme of deleting exon 3 of *Exd2* with a pair of gRNAs.
(B) Genomic DNA PCR analysis of knockout of exon 3 with indicated 5' primer and 3' primer.
(C) Western blot analysis of CRISPR/Cas9-mediated knockout of Exd2 in WT and *Lig4*$^{-/-}$ cells. Two positive deletion clones were obtained in each background.
(D) Normalized IgA switching efficiency in *Exd2* deleted WT and *Lig4*$^{-/-}$ cells measured by FACS. Data were presented as mean ± SD from four independent experiments (Student's t-test, *p < 0.05, n.s. (p>0.05) indicates non-significant differences).

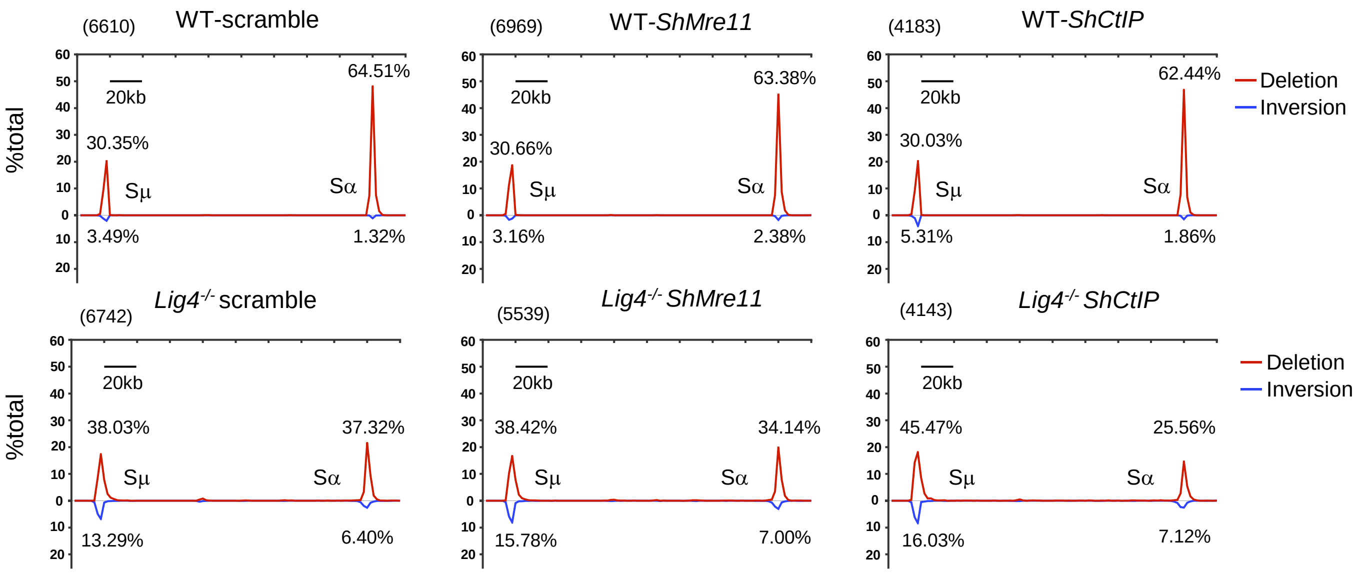

Supplementary Figure S4. HTGTS analysis of Mre11/CtIP-silenced WT and *Lig4*$^{-/-}$ cells
Linear distribution of pooled junctions recovered from CSR activated Mre11/CtIP-silenced WT and *Lig4*$^{-/-}$ cells with at least three experiment repeats each are shown in the forms of deletion or inversion along a 200kb region across *IgH* locus (Chr12: 114480001-114680000). Bin size is 20kb and 100 bins are presented in each plot. Numbers in the parenthesis represent total unique junctions in the indicated regions.

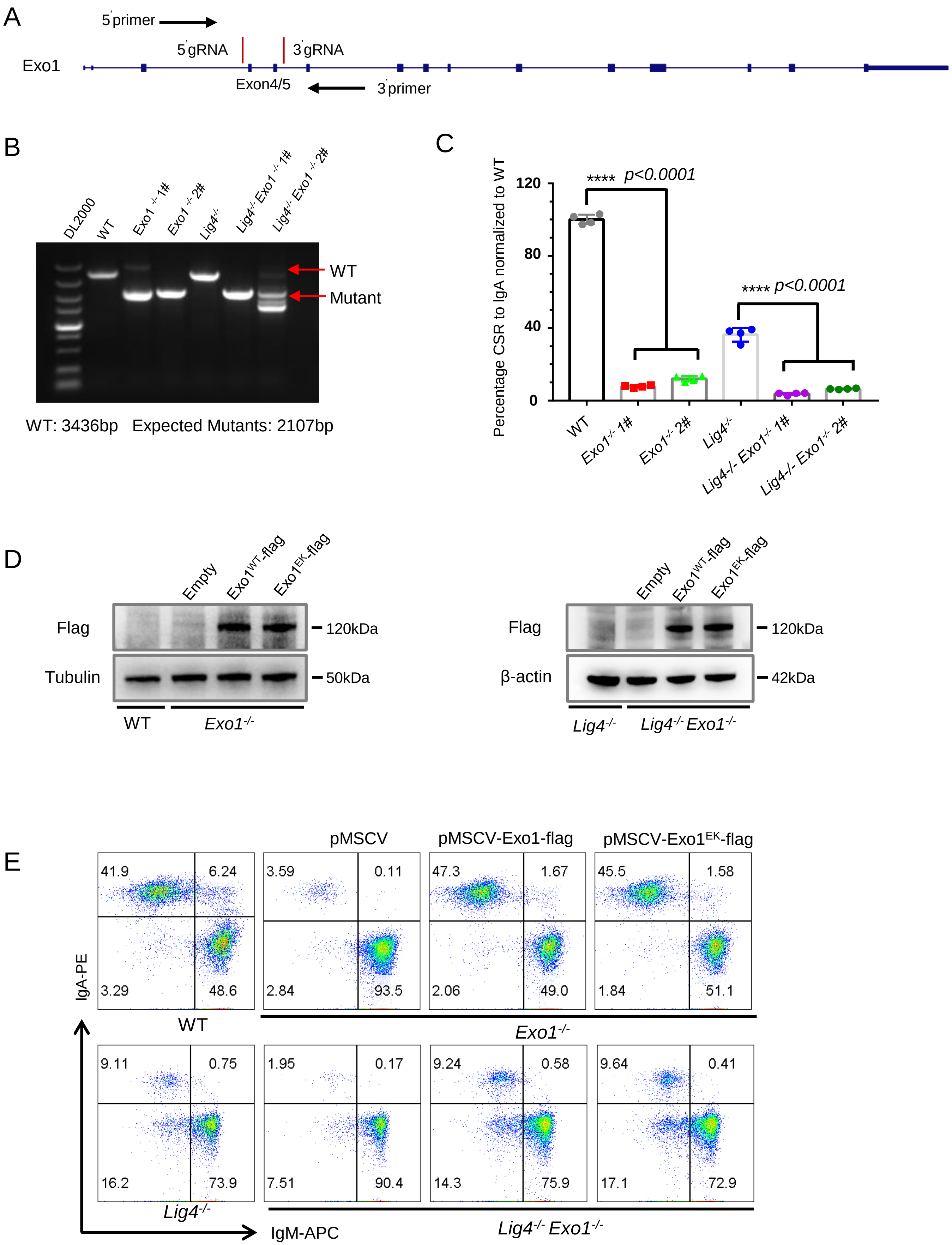

A
5′primer
5′gRNA
3′gRNA
Exo1
Exon4/5
3′primer
B
DL2000
WT
Exo1 -/- 1#
Exo1 -/- 2#
Lig4-/-
Lig4-/- Exo1 -/- 1#
Lig4-/- Exo1 -/- 2#
WT
Mutant
WT: 3436bp Expected Mutants: 2107bp
C
Percentage CSR to IgA normalized to WT
**** p<0.0001
**** p<0.0001
120
80
40
0
WT
Exo1-/- 1#
Exo1-/- 2#
Lig4-/-
Lig4-/- Exo1-/- 1#
Lig4-/- Exo1-/- 2#
D
Empty
Exo1WT-flag
Exo1EK-flag
Flag
Tubulin
120kDa
50kDa
WT
Exo1-/-
Flag
β-actin
120kDa
42kDa
Lig4-/-
Lig4-/- Exo1-/-
E
pMSCV
pMSCV-Exo1-flag
pMSCV-Exo1EK-flag
41.9
6.24
3.29
48.6
3.59
0.11
2.84
93.5
47.3
1.67
2.06
49.0
45.5
1.58
1.84
51.1
IgA-PE
WT
Exo1-/-
9.11
0.75
16.2
73.9
1.95
0.17
7.51
90.4
9.24
0.58
14.3
75.9
9.64
0.41
17.1
72.9
Lig4-/-
IgM-APC
Lig4-/- Exo1-/-

F

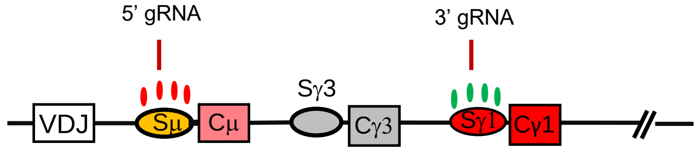

G

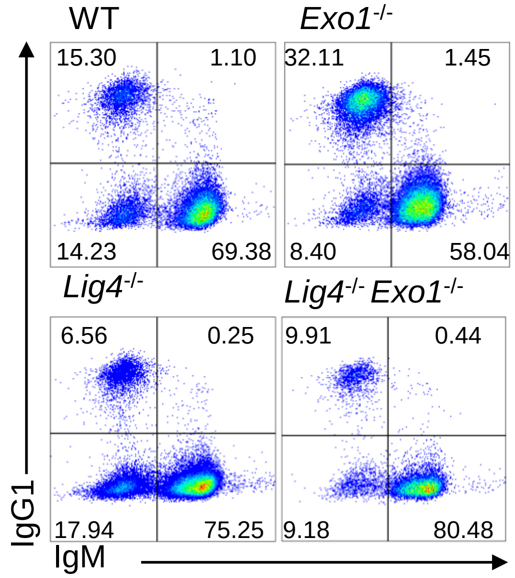

H

GFP+(%)	24h	48h	72h
WT 1	32.49	11.70	1.13
WT 2	31.41	10.41	0.97
WT 3	30.88	10.16	0.93
Exo1$^{-/-}$ 1	54.09	38.62	8.12
Exo1$^{-/-}$ 2	53.81	38.93	7.67
Exo1$^{-/-}$ 3	54.52	35.86	7.91
Lig4$^{-/-}$ 1	21.75	9.31	0.97
Lig4$^{-/-}$ 2	22.28	11.19	1.01
Lig4$^{-/-}$ 3	24.78	9.52	0.75
Lig4$^{-/-}$ *Exo1*$^{-/-}$ 1	48.06	25.49	4.62
Lig4$^{-/-}$ *Exo1*$^{-/-}$ 2	48.60	31.85	5.63
Lig4$^{-/-}$ *Exo1*$^{-/-}$ 3	44.92	31.67	4.79

Supplementary Figure S5. The nuclease of Exo1 is not required for AID-initiated and CRISPR/Cas9 induced CSR

(A)The scheme of deleting exons 4 and 5 of Exo1 with a pair of gRNAs in WT and *Lig4*$^{-/-}$ cells.

(B) Genomic DNA PCR analysis of knockout of exons 4-5 with indicated 5' primer and 3' primer, two positive knockout clones in each background were obtained.

(C) Normalized switching efficiency to lgA in *Exo1* deleted WT and *Lig4*$^{-/-}$ cells. Data were presented as mean ± SD from four independent experiments (Student's t-test, ****p < 0.0001, n.s. (p>0.05) indicates non-significant differences).

(D) Western blot analysis of Exo1-deficient WT and *Lig4*$^{-/-}$ cells reconstituted with retrovirus expressing indicated constructs.

(E)Representative flow cytometry analysis of switching to lgA in *Exo1*$^{-/-}$ and *Lig4*$^{-/-}$ *EXO1*$^{-/-}$ cells transduced with retrovirus expression vector expressing Exo1-Flag or Exo1^{EK}-Flag.

(F) The joining assay with CRISPR/gRNAs targeting Sμ and Sγ1 locus multiple times.

(G) Representative flow cytometry analysis result of Sμ-CRISPR/Cas9 and Sγ1-CRISPR/Cas9-mediated switching to lgG1 in Exo1-deficient WT and Lig4$^{-/-}$ cells measured 72h after transfection.

(H) GFP positive ratio measure 24h, 48h and 72h post CRISPR/Cas9 plasmids nucleofection in Exo1-deficient WT and *Lig4*$^{-/-}$ cells.

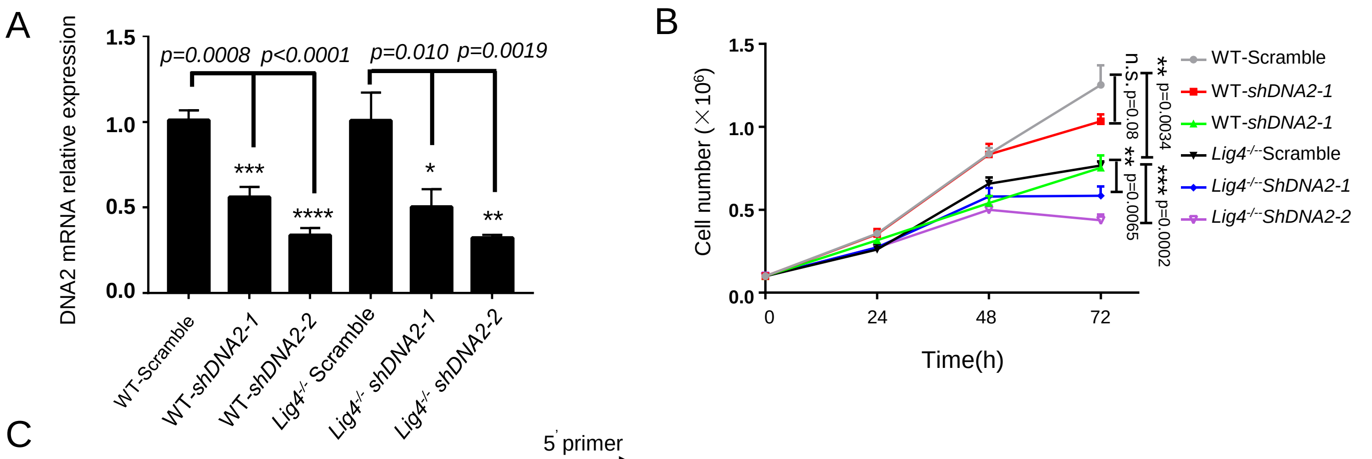

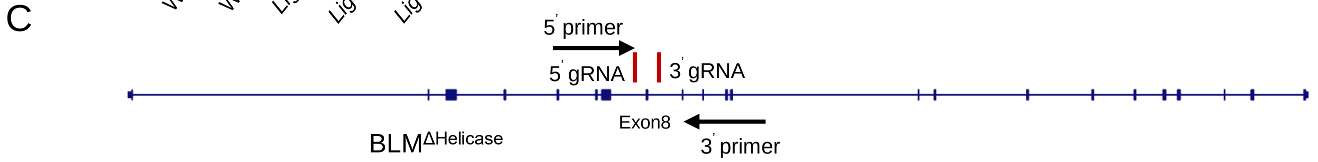

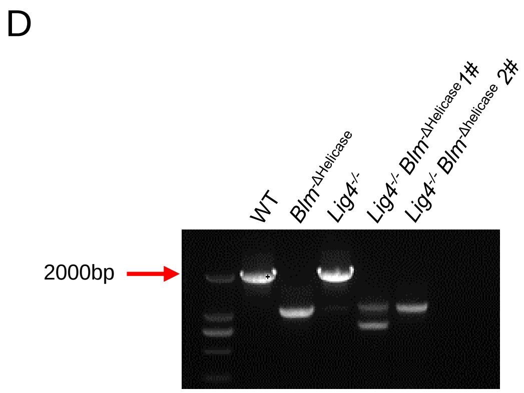

Genotype	Allele-1(deletion)	Allele-1(deletion)
Blm$^{\Delta Helicase}$	979bp	1029bp
Lig4$^{-/-}$ *Blm*$^{\Delta Helicase}$1#	996bp	1278bp
Lig4$^{-/-}$ *Blm*$^{\Delta Helicase}$2#	988bp	991bp

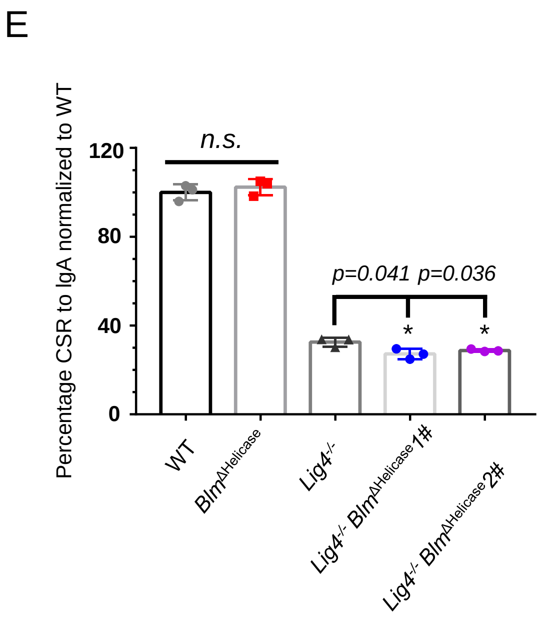

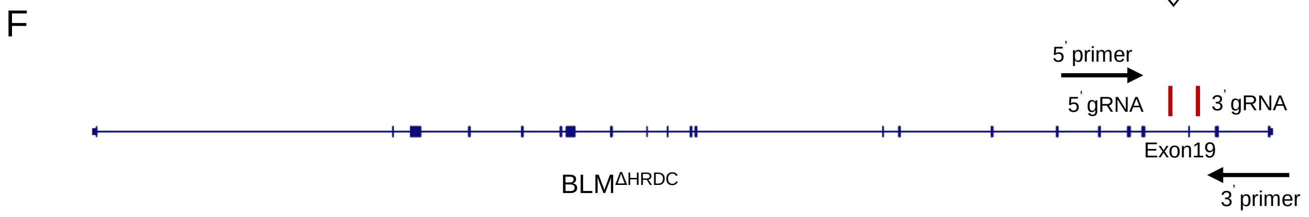

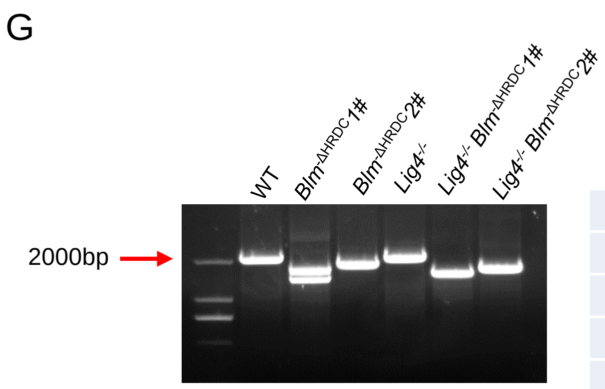

Genotype	Allele-1(deletion)	Allele-2(deletion)
Blm$^{\Delta HRDC}$1#	731bp	411bp
Blm$^{\Delta HRDC}$2#	425bp	255bp
Lig4$^{-/-}$ Blm$^{\Delta HRDC}$1#	597bp	
Lig4$^{-/-}$ Blm$^{\Delta HRDC}$2#	453bp	550bp

H

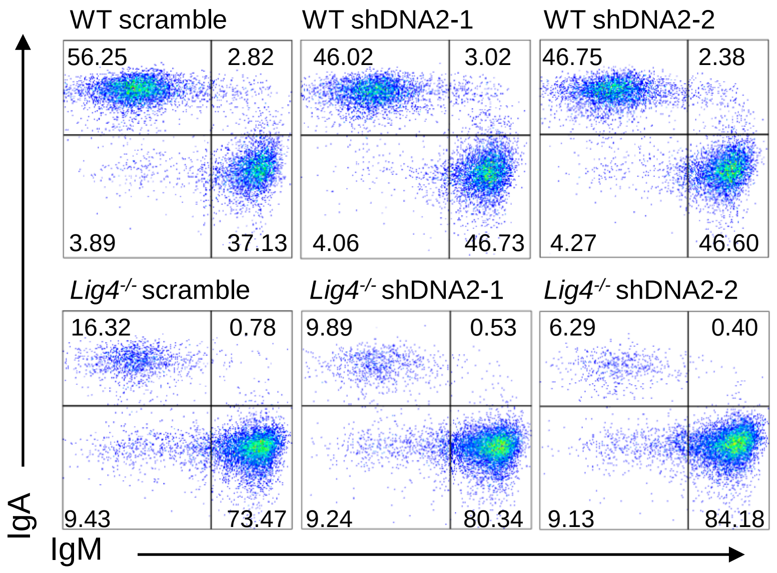
WT scramble
WT shDNA2-1
WT shDNA2-2
56.25
2.82
3.89
37.13
46.02
3.02
4.06
46.73
46.75
2.38
4.27
46.60
Lig4-/- scramble
Lig4-/- shDNA2-1
Lig4-/- shDNA2-2
16.32
0.78
9.43
73.47
9.89
0.53
9.24
80.34
6.29
0.40
9.13
84.18
IgA
IgM

I

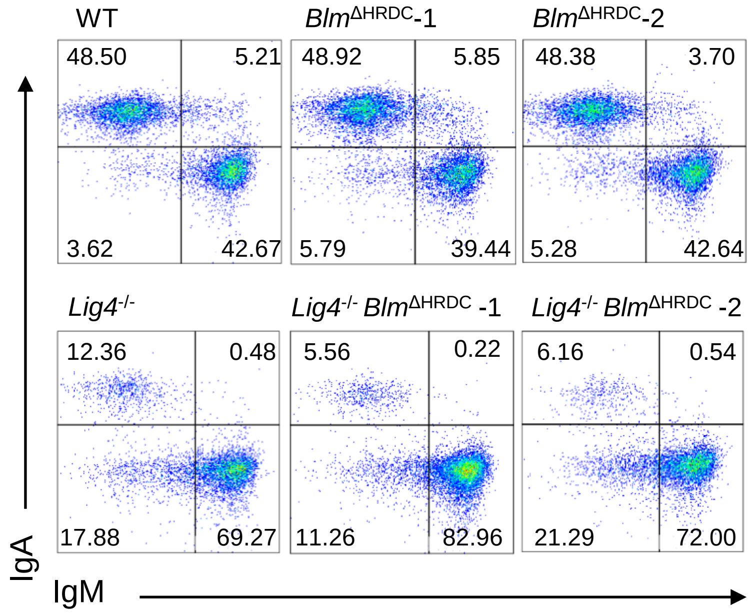
WT
BlmΔHRDC-1
BlmΔHRDC-2
48.50
5.21
3.62
42.67
48.92
5.85
5.79
39.44
48.38
3.70
5.28
42.64
Lig4-/-
Lig4-/- BlmΔHRDC -1
Lig4-/- BlmΔHRDC -2
12.36
0.48
17.88
69.27
5.56
0.22
11.26
82.96
6.16
0.54
21.29
72.00
IgA
IgM

Supplementary Figure S6. DNA2 and helicase activity of BLM in A-EJ-mediated CSR

(A) qRT-PCR analysis of DNA2 mRNA expression in WT and *Lig4*$^{-/-}$ cells transduced with indicated shDNA2 lentivirus. Data were presented as mean ± SD from three independent experiments (Student's t-test, *p < 0.05, **p < 0.01, ***p < 0.001, ****p < 0.0001, n.s. (p>0.05) indicates non-significant differences).

(B) The proliferation curves of WT and *Lig4*$^{-/-}$ cells transduced with Scramble or shDNA2 lentivirus stimulated with αCD40/IL-4/TGF-β. The cells were collected at 0, 24h, 48h and 72h after stimulation, stained with trypan blue and counted with hemacytometer. Data were presented as mean ± SD from three independent experiments (Student's t-test, *p < 0.05, **p < 0.01，***p < 0.001，n.s. (p>0.05) indicates non-significant differences).

(C) The scheme of deleting BLM helicase domain with a pair of gRNAs flanking exon 8.

(D) Genomic DNA PCR analysis of Blm helicase domain deleted with indicated 5' primer and 3' primer and the results of T-A cloning and sequencing analysis of junctions by deleting exon 8 of *Blm* with a pair of CRISPR-Cas9 gRNAs.

(E) Normalized IgA switching efficiency in Blm helicase domain deleted WT and *Lig4*$^{-/-}$ cells. Data were presented as mean ± SD from three independent experiments (Student's t-test, *p < 0.05, n.s. (p>0.05) indicates non-significant differences).

(F) The scheme of deleting HRDC domain of BLM with a pair of gRNAs flanking exon 19.

(G)Genomic DNA PCR analysis of HRDC domain deletion with indicated 5' primer and 3' primer (left). The results Sanger sequencing of PCR products of exon19 deletion junctions by Cas9 with indicated primers are shown (right).

(H) Representative flow cytometry analysis result of scramble and shDNA2 lenti-virus transduction in WT and Lig4$^{-/-}$ background cells after 72h CIT stimulation.

(I) Representative flow cytometry analysis result of BLM ΔHRDC in WT and *Lig4*$^{-/-}$ background cells after 72h CIT stimulation.

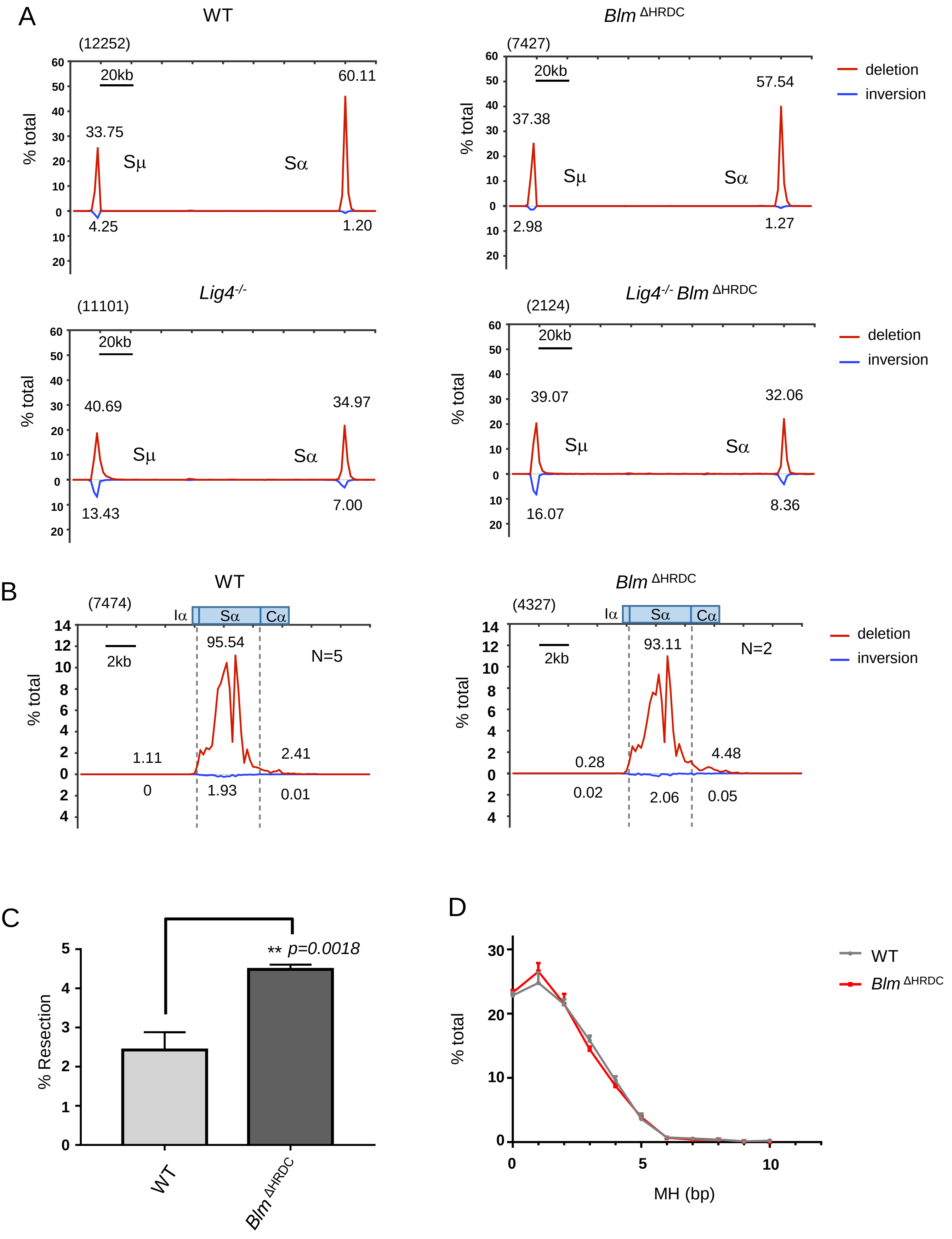

A
WT
(12252)
20kb
33.75
60.11
Sμ
Sα
4.25
1.20
% total
Blm ΔHRDC
(7427)
20kb
37.38
57.54
Sμ
Sα
2.98
1.27
deletion
inversion
Lig4-/-
(11101)
20kb
40.69
34.97
Sμ
Sα
13.43
7.00
Lig4-/- Blm ΔHRDC
(2124)
20kb
39.07
32.06
Sμ
Sα
16.07
8.36
deletion
inversion
B
WT
(7474)
Iα
Sα
Cα
95.54
2kb
N=5
1.11
2.41
0
1.93
0.01
Blm ΔHRDC
(4327)
Iα
Sα
Cα
93.11
2kb
N=2
0.28
4.48
0.02
2.06
0.05
deletion
inversion
C
** p=0.0018
% Resection
WT
Blm ΔHRDC
D
% total
WT
Blm ΔHRDC
MH (bp)

Supplementary Figure S7. HTGTS profile of HRDC domain of BLM-deleted WT and *Lig4*$^{-/-}$ cells

(A) Linear distribution of pooled junctions recovered from CSR activated WT and *Lig4*$^{-/-}$ cells with HRDC domain of BLM deleted were shown in the forms of deletion or inversion along a 200kb region across *IgH* locus.

(B) Linear distribution of pooled Sμ-Sα junctions recovered from HTGTS libraries with CSR activated BLM HRDC domain deleted WT cells.

(C) Percentage of long resection junctions recovered from HTGTS libraries with CSR activated *Blm*$^{\Delta HRDC}$ cells. Data were presented as mean ± SEM. (Student's t-test, **$p < 0.01$, n.s. ($p>0.05$) indicates non-significant differences).

(D) The MH pattern of Sμ-Sα junctions in CSR activated *Blm*$^{\Delta HRDC}$ cells. Data were presented as mean ± SEM. (Student's t-test).

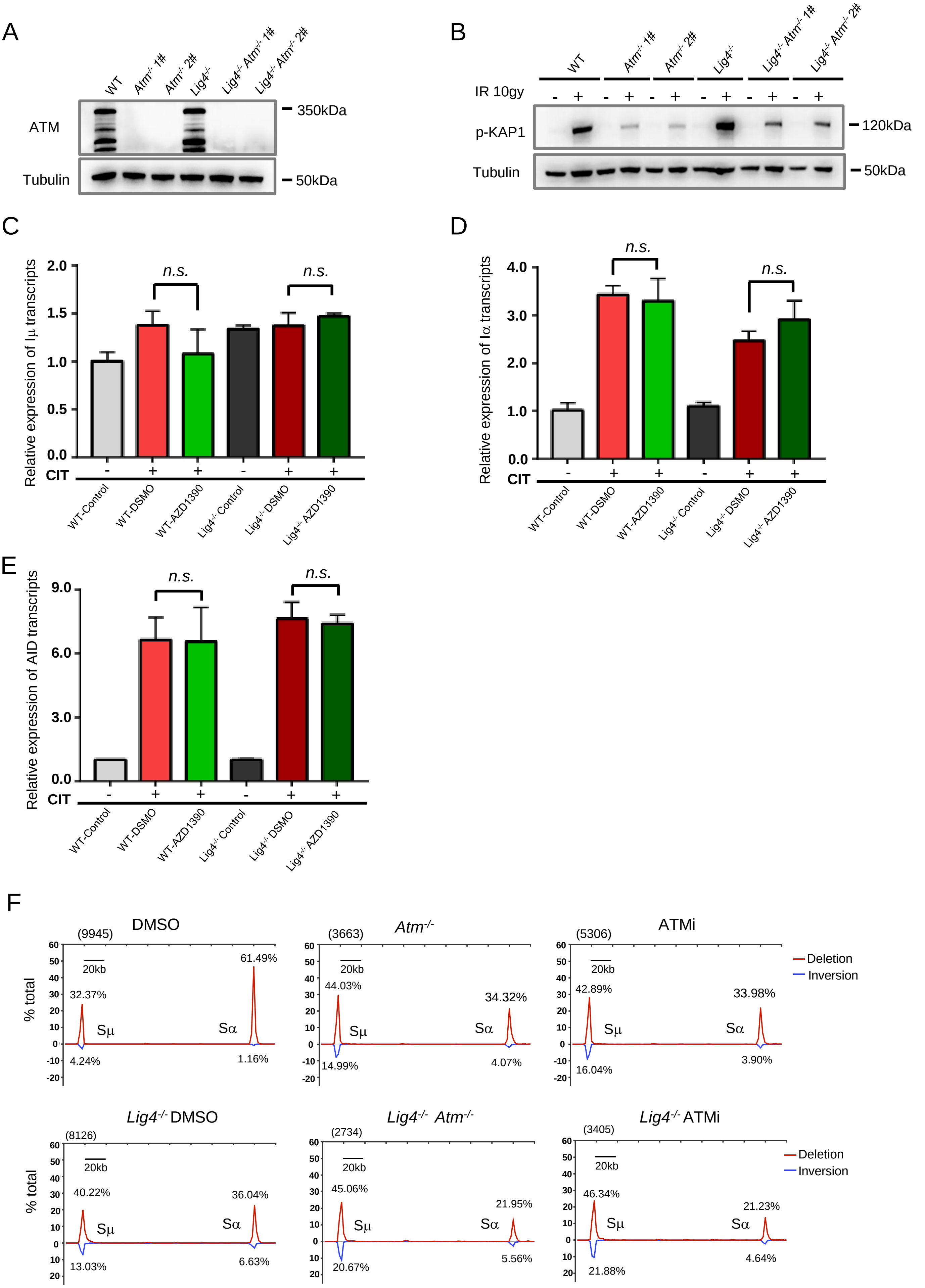
A
WT
Atm-/- 1#
Atm-/- 2#
Lig4-/-
Lig4-/- Atm-/- 1#
Lig4-/- Atm-/- 2#
ATM
350kDa
Tubulin
50kDa
B
WT
Atm-/- 1#
Atm-/- 2#
Lig4-/-
Lig4-/- Atm-/- 1#
Lig4-/- Atm-/- 2#
IR 10gy
p-KAP1
120kDa
Tubulin
50kDa
C
Relative expression of Iμ transcripts
n.s.
n.s.
CIT
WT-Control
WT-DSMO
WT-AZD1390
Lig4-/- Control
Lig4-/- DSMO
Lig4-/- AZD1390
D
Relative expression of Iα transcripts
n.s.
n.s.
CIT
WT-Control
WT-DSMO
WT-AZD1390
Lig4-/- Control
Lig4-/- DSMO
Lig4-/- AZD1390
E
Relative expression of AID transcripts
n.s.
n.s.
CIT
WT-Control
WT-DSMO
WT-AZD1390
Lig4-/- Control
Lig4-/- DSMO
Lig4-/- AZD1390
F
DMSO
(9945)
Atm-/-
(3663)
ATMi
(5306)
% total
20kb
Sμ
Sα
32.37%
61.49%
4.24%
1.16%
44.03%
34.32%
14.99%
4.07%
42.89%
33.98%
16.04%
3.90%
Deletion
Inversion
Lig4-/- DMSO
(8126)
Lig4-/- Atm-/-
(2734)
Lig4-/- ATMi
(3405)
40.22%
36.04%
13.03%
6.63%
45.06%
21.95%
20.67%
5.56%
46.34%
21.23%
21.88%
4.64%

G

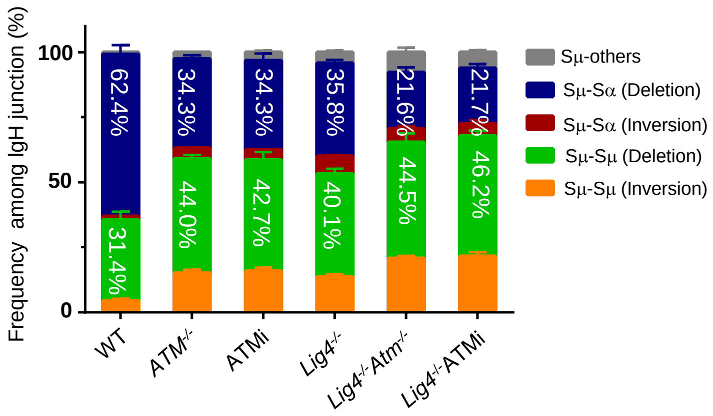

H

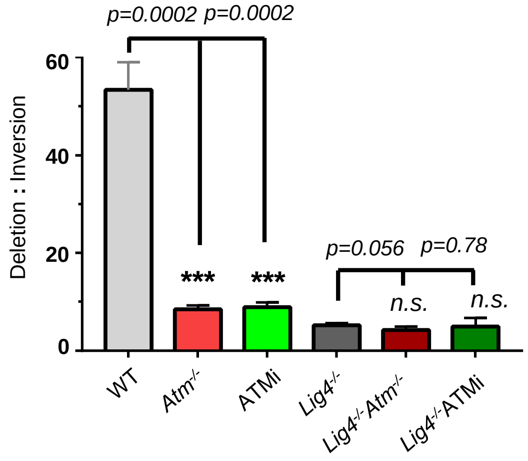

Supplementary Figure S8. HTGTS profile of *Atm*-deleted WT and *Lig4*$^{-/-}$ cells

(A)Western blot analysis of ATM expression in WT and *Lig4*$^{-/-}$ cells with CRISPR/Cas9-mediated deletion of exons 59-60 of *Atm*.

(B)Western blot analysis of phospho-KAP1 expression in knockout of ATM WT and *Lig4*$^{-/-}$ cells irradiated with 0 or 10 gy IR. The cell lysates were collected 2h after IR for western blot analysis.

(C-D) qRT-PCR analysis of spliced mature Iμ-Cμ and Iα-Cα germline transcripts in WT and *Lig4*$^{-/-}$ cells pretreated with DMSO or 100nM AZD1390 without (-) or with (+) αCD40/IL-4/TGF-β stimulation for 16h. Data were presented as mean ± SD from three independent experiments (Student's t-test, *p < 0.05, n.s. (p>0.05) indicates non-significant differences).

(E) qRT-PCR analysis of AID mRNA levels in WT and *Lig4*$^{-/-}$ cells pretreated with DMSO or 100nM AZD1390 without (-) or with (+) αCD40/IL-4/TGF-β stimulation for 16h. Data were presented as mean ± SD from three independent experiments (Student's t-test, *p < 0.05, n.s. (p>0.05) indicates non-significant differences).

(F) Linear distribution of pooled junctions recovered from CSR activated *Atm*-deleted or AZD1390-treated WT and *Lig4*$^{-/-}$ cells with three experiment replicates each are shown in the forms of deletion or inversion along a 200kb region across *IgH* locus (Chr12: 114480001-114680000). Bin size is 20kb and 100 bins are presented in each plot. Numbers in the parenthesis represent total unique junctions in the indicated regions.

(G) Distribution of junctions within *IgH* locus in CSR activated ATM deleted or inhibited WT and *Lig4*$^{-/-}$ cells. The percentage of Sμ-Sμ and Sμ-Sα joining in either orientation in indicated backgrounds were shown. Data were presented as mean ± SEM. (Student's t-test, n.s. (p>0.05) indicates non-significant differences).

(H) The ratio of deletion versus inversion for Sα junctions in CSR activated *Atm* knockout and kinase inhibitor treated WT and *Lig4*$^{-/-}$ cells. Data were presented as mean ± SEM. (Student's t-test, ***p < 0.001, n.s. (p>0.05) indicates non-significant differences).

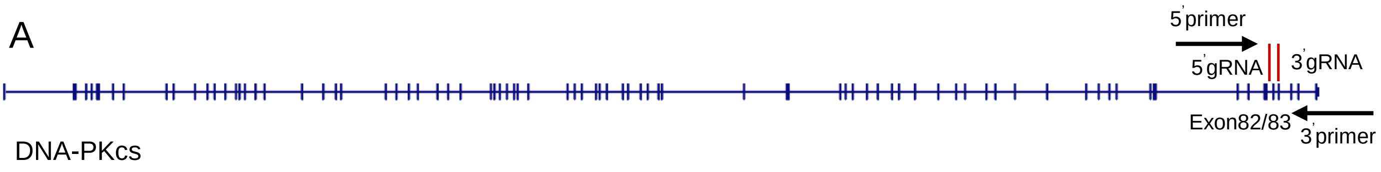

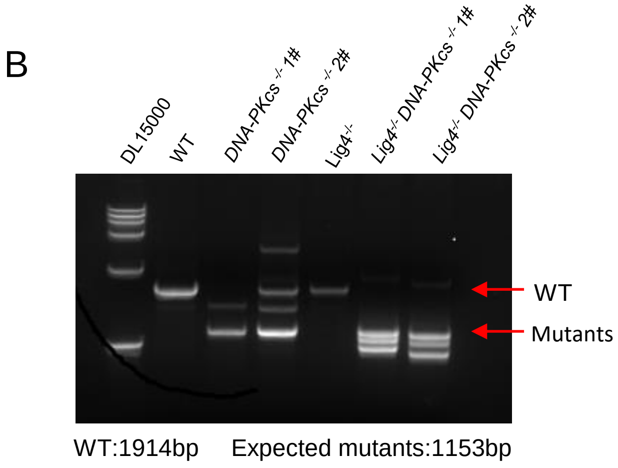

C

Genotype	Allele-1(deletion)	Allele-2
DNA-PKcs $^{-/-}$ 1#	765bp	343bp(deletion)
DNA-PKcs $^{-/-}$ 2#	759bp	787bp(inversion)
Lig4 $^{-/-}$ *DNA-PKcs* $^{-/-}$ 1#	772bp	962bp(deletion)
Lig4 $^{-/-}$ *DNA-PKcs* $^{-/-}$ 2#	795bp	1018bp(deletion)

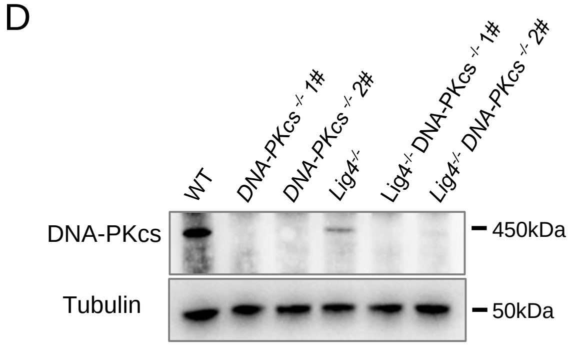

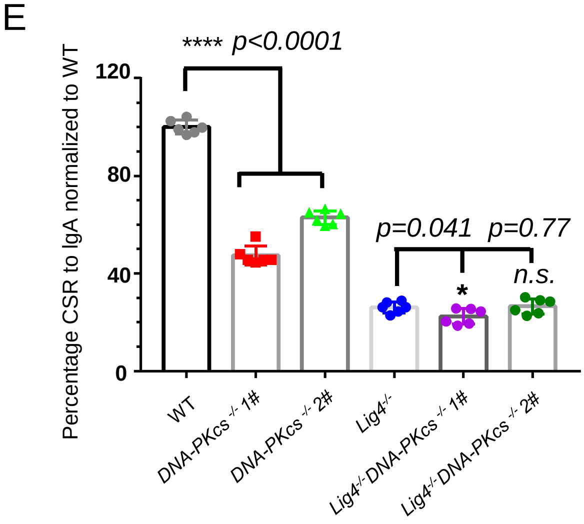

Supplementary Figure S9. DNA-PKcs is required for only c-NHEJ mediated CSR

(A)The scheme of deleting exons 82 and 83 of *Prkdc* gene with a pair of CRISPR-Cas9 gRNAs.

(B) Genomic DNA PCR analysis of knockout of exons 82 and 83 of *Prkdc* with indicated 5’ and 3’ primers.

(C) Sequencing analysis of junctions by deleting exons 82 and 83 of *Prkdc* with a pair of CRISPR-Cas9 gRNA. The genomic DNA surrounding *Prkdc* exons 82 and 83 were amplified by PCR with indicated 5’ primer and 3’ primer. PCR products were subjected to T-A clone and Sanger sequencing for confirmation.

(D) Western blot analysis of DNA-PKcs expression in WT and *Lig4* $^{-/-}$ cells with CRISPR/Cas9-mediated deletion of *Prkdc*.

(E) Normalized IgA switching in *Prkdc* exons 82-83 deleted WT and *Lig4* $^{-/-}$ cells measured by FACS at 72h post stimulation with αCD40/IL-4/TGF-β. Data were presented as mean ± SD from six independent experiments (Student’s t-test, *p < 0.05, ***p < 0.001, n.s. (p>0.05) indicates non-significant differences).

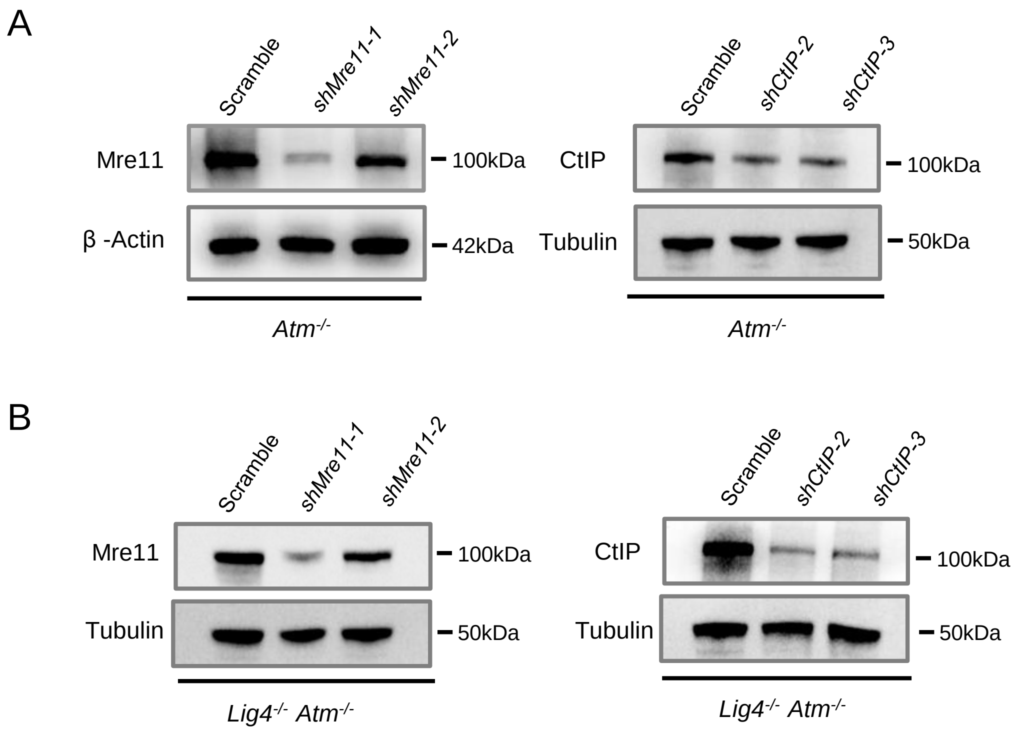

Supplementary Figure S10. knockdown of Mre11/CtIP in ATM-deficient B cells

(A)Western blot analysis of Mre11/CtIP expression in *Atm*$^{-/-}$ cells transduced with lentivirus expressing the indicated shRNAs.

(B) Western blot analysis of Mre11/CtIP expression in *Lig4*$^{-/-}$ *Atm*$^{-/-}$ cells transduced with lentivirus expressing the indicated shRNAs.

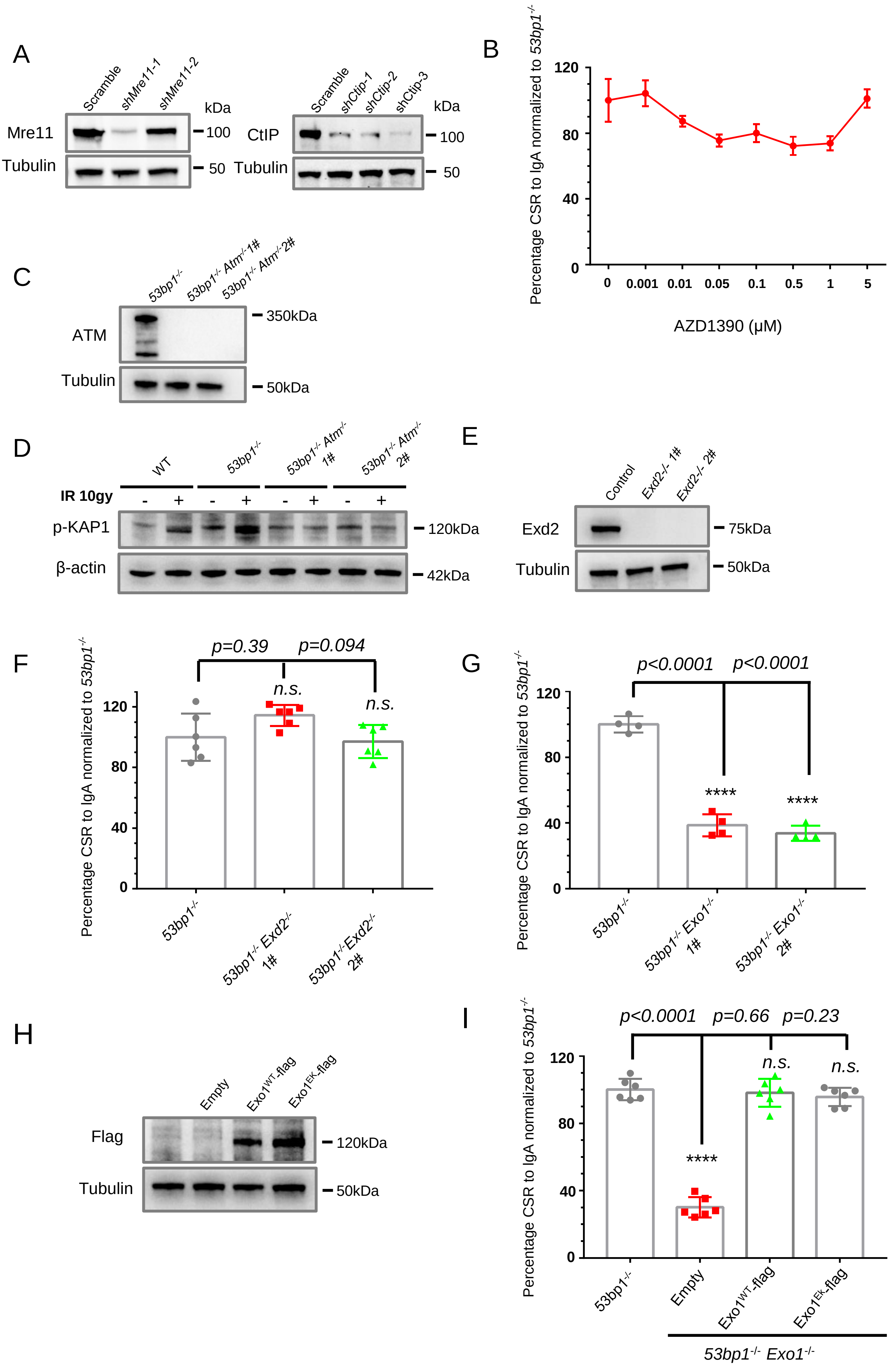
A
Scramble
shMre11-1
shMre11-2
kDa
Mre11
100
Tubulin
50
Scramble
shCtip-1
shCtip-2
shCtip-3
kDa
CtIP
100
Tubulin
50
B
Percentage CSR to IgA normalized to 53bp1-/-
120
80
40
0
0
0.001
0.01
0.05
0.1
0.5
1
5
AZD1390 (μM)
C
53bp1-/-
53bp1-/- Atm-/-1#
53bp1-/- Atm-/-2#
ATM
350kDa
Tubulin
50kDa
D
WT
53bp1-/-
53bp1-/- Atm-/- 1#
53bp1-/- Atm-/- 2#
IR 10gy
- + - + - + - +
p-KAP1
120kDa
β-actin
42kDa
E
Control
Exd2-/- 1#
Exd2-/- 2#
Exd2
75kDa
Tubulin
50kDa
F
Percentage CSR to IgA normalized to 53bp1-/-
p=0.39
p=0.094
n.s.
n.s.
120
80
40
0
53bp1-/-
53bp1-/- Exd2-/- 1#
53bp1-/- Exd2-/- 2#
G
Percentage CSR to IgA normalized to 53bp1-/-
p<0.0001
p<0.0001
****
****
120
80
40
0
53bp1-/-
53bp1-/- Exo1-/- 1#
53bp1-/- Exo1-/- 2#
H
Empty
Exo1WT-flag
Exo1EK-flag
Flag
120kDa
Tubulin
50kDa
I
Percentage CSR to IgA normalized to 53bp1-/-
p<0.0001
p=0.66
p=0.23
n.s.
n.s.
****
120
80
40
0
53bp1-/-
Empty
Exo1WT-flag
Exo1EK-flag
53bp1-/- Exo1-/-

Supplementary Figure S11. Effect of resection factors on A-EJ mediated CSR in *53bp1*$^{-/-}$ cells

(A)Western blot analysis of Mre11/CtIP expression in *53bp1*$^{-/-}$ cells transduced with lentivirus expressing the indicated shRNAs.

(B) Normalized lgA switching efficiency in *53bp1*$^{-/-}$ cells treated with AZD1390 at concentrations ranging from 0μM, 0.001μM, 0.01μM, 0.05μM, 0.1μM, 0.5μM, 1μM to 5μM. Data were presented as mean ± SD from three independent experiments (Student's t-test).

(C) Western blot of ATM expression in CRISPR/Cas9-mediated deletion of *Atm* in *53bp1* $^{-/-}$ cells.

(D) Western blot analysis of phospho-KAP1 expression in *53bp1*$^{-/-}$ *Atm*$^{-/-}$ cells irradiated with 0 or 10 gy IR. The cell lysates were collected 2h after IR.

(E) Western blot analysis of Exd2 expression in *53bp1*$^{-/-}$ cells with CRISPR/Cas9 mediated deletion of *Exd2*.

(F) Normalized switching to lgA in *53bp1*$^{-/-}$ *Exd2*$^{-/-}$ cells examined by FACS with α-CD40/IL-4/TGF-β. Data were presented as mean ± SD from six independent experiments (Student's t-test, *p < 0.05, n.s. (p>0.05) indicates non-significant differences).

(G) Percentage of CSR switching to lgA in *53bp1*$^{-/-}$ *Exo1*$^{-/-}$ cell lines. Data were presented as mean ± SD from four independent experiments (Student's t-test, ****p < 0.0001, n.s. (p>0.05) indicates non-significant differences).

(H) Western blot analysis of flag-tagged proten expression in 53bp1 and Exo1 double-deficient B cell reconstituted with retrovirus expressing indicated constructs.

(I) Percentage of CSR switching to lgA in *53bp1*$^{-/-}$ *Exo1*$^{-/-}$ cell lines reconstituted with retrovirus expressing indicated constructs. Data were presented as mean ± SD from six independent experiments (Student's t-test, *p < 0.05, ****p < 0.0001, n.s. (p>0.05) indicates non-significant differences).

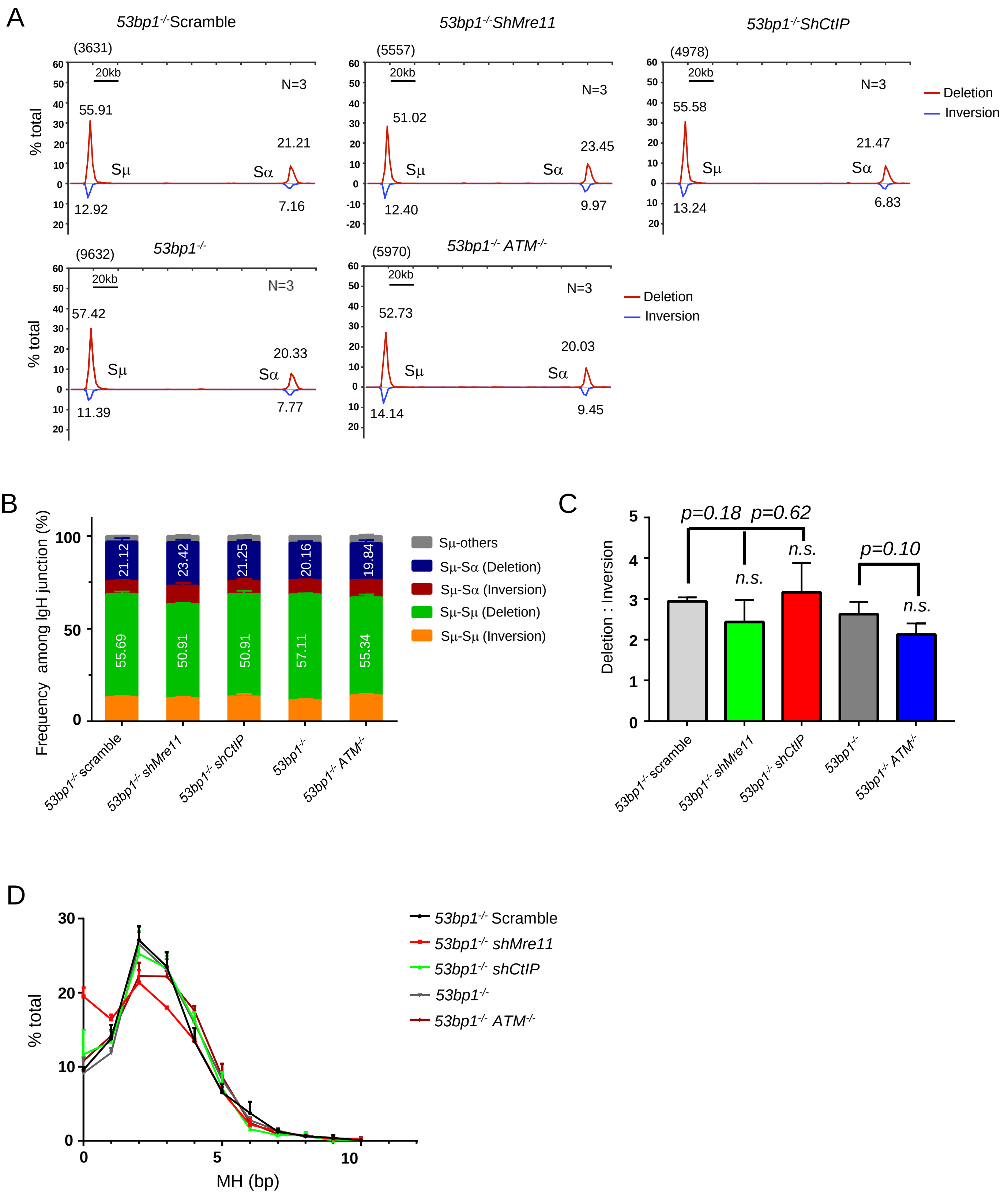

A
53bp1-/-Scramble
53bp1-/-ShMre11
53bp1-/-ShCtIP
53bp1-/-
53bp1-/- ATM-/-
(3631)
(5557)
(4978)
(9632)
(5970)
20kb
N=3
% total
55.91
21.21
12.92
7.16
51.02
23.45
12.40
9.97
55.58
21.47
13.24
6.83
57.42
20.33
11.39
7.77
52.73
20.03
14.14
9.45
Sμ
Sα
Deletion
Inversion
B
Frequency among IgH junction (%)
21.12
23.42
21.25
20.16
19.84
55.69
50.91
50.91
57.11
55.34
Sμ-others
Sμ-Sα (Deletion)
Sμ-Sα (Inversion)
Sμ-Sμ (Deletion)
Sμ-Sμ (Inversion)
53bp1-/- scramble
53bp1-/- shMre11
53bp1-/- shCtIP
53bp1-/-
53bp1-/- ATM-/-
C
Deletion : Inversion
p=0.18
p=0.62
p=0.10
n.s.
n.s.
n.s.
53bp1-/- scramble
53bp1-/- shMre11
53bp1-/- shCtIP
53bp1-/-
53bp1-/- ATM-/-
D
% total
MH (bp)
53bp1-/- Scramble
53bp1-/- shMre11
53bp1-/- shCtIP
53bp1-/-
53bp1-/- ATM-/-

Supplementary Figure S12. HTGTS analysis of Mre11/CtIP/ATM ablated *53bp1*$^{-/-}$ cells

(A) Linear distribution of pooled junctions recovered from CSR activated shMre11 or shCtIP transduced or *Atm* deleted *53bp1*$^{-/-}$ cells with three experiment repeats each are shown in the forms of deletion or inversion along a 200kb region across *IgH* locus (Chr12: 114480001-114680000). Bin size is 20kb and 100 bins are presented in each plot. Numbers in the parenthesis represent total unique junctions in the indicated regions.

(B) Junctions distribution within *IgH* locus in CSR activated Mre11 or CtIP-silenced and *Atm*-deleted *53bp1*$^{-/-}$ cells. The percentage of Sμ-Sμ and Sμ-Sα joining in either orientation in indicated backgrounds were shown. Data were presented as mean ± SEM. (Student's t-test).

(C) The ratio of deletion versus inversion for Sα junctions in CSR activated Mre11/CtIP silenced and *Atm*-deleted *53bp1*$^{-/-}$ cells. Data were presented as mean ± SEM. (Student's t-test, *$p < 0.05$, n.s. ($p>0.05$) indicates non-significant differences).

(D) MH pattern in Sμ-Sα junctions recovered from HTGTS libraries with CSR activated Mre11/CtIP-silenced and ATM deficient *53bp1*$^{-/-}$ cells. Data were presented as mean ± SEM. (Student's t-test)
